# Compassion Focused Group Therapy for People With a Diagnosis of Bipolar Affective Disorder: A Feasibility Study

**DOI:** 10.3389/fpsyg.2022.841932

**Published:** 2022-07-20

**Authors:** Paul Gilbert, Jaskaran K. Basran, Joanne Raven, Hannah Gilbert, Nicola Petrocchi, Simone Cheli, Andrew Rayner, Alison Hayes, Kate Lucre, Paschalina Minou, David Giles, Frances Byrne, Elizabeth Newton, Kirsten McEwan

**Affiliations:** ^1^Centre for Compassion Research and Training, College of Health, Psychology and Social Care, University of Derby, Derby, United Kingdom; ^2^The Compassionate Mind Foundation, Derby, United Kingdom; ^3^Department of Psychology, University of Roehampton, London, United Kingdom; ^4^Department of Economics and Social Sciences, John Cabot University, Rome, Italy; ^5^Compassionate Mind ITALIA, Rome, Italy; ^6^School of Human Health Sciences, University of Florence, Florence, Italy; ^7^Birmingham and Solihull Mental Health NHS Foundation Trust, Birmingham, United Kingdom; ^8^Department of Philosophy, University College London, London, United Kingdom; ^9^College of Health, Psychology and Social Care, University of Derby, Derby, United Kingdom; ^10^Lattice Coaching and Training, Chesterfield, United Kingdom

**Keywords:** bipolar, compassion focused therapy, competitiveness, heart rate variability, biopsychosocial, caring

## Abstract

**Background:**

Compassion focused therapy (CFT) is an evolutionary informed, biopsychosocial approach to mental health problems and therapy. It suggests that evolved motives (e.g., for caring, cooperating, competing) are major sources for the organisation of psychophysiological processes which underpin mental health problems. Hence, evolved motives can be targets for psychotherapy. People with certain types of depression are psychophysiologically orientated towards social competition and concerned with social status and social rank. These can give rise to down rank-focused forms of social comparison, sense of inferiority, worthlessness, lowered confidence, submissive behaviour, shame proneness and self-criticism. People with bipolar disorders also experience elevated aspects of competitiveness and up rank status evaluation. These shift processing to a sense of superiority, elevated confidence, energised behaviour, positive affect and social dominance. This is the first study to explore the feasibility of a 12 module CFT group, tailored to helping people with a diagnosis of bipolar disorder understand the impact of evolved competitive, status-regulating motivation on their mental states and the value of cultivating caring and compassion motives and their psychophysiological regulators.

**Methods:**

Six participants with a history of bipolar disorder took part in a CFT group consisting of 12 modules (over 25 sessions) as co-collaborators to explore their personal experiences of CFT and potential processes of change. Assessment of change was measured via self-report, heart rate variability (HRV) and focus groups over three time points.

**Results:**

Although changes in self-report scales between participants and across time were uneven, four of the six participants consistently showed improvements across the majority of self-report measures. Heart rate variability measures revealed significant improvement over the course of the therapy. Qualitative data from three focus groups revealed participants found CFT gave them helpful insight into: how evolution has given rise to a number of difficult problems for emotion regulation (called tricky brain) which is not one’s fault; an evolutionary understanding of the nature of bipolar disorders; development of a compassionate mind and practices of compassion focused visualisations, styles of thinking and behaviours; addressing issues of self-criticism; and building a sense of a compassionate identity as a means of coping with life difficulties. These impacted their emotional regulation and social relationships.

**Conclusion:**

Although small, the study provides evidence of feasibility, acceptability and engagement with CFT. Focus group analysis revealed that participants were able to switch from competitive focused to compassion focused processing with consequent improvements in mental states and social behaviour. Participants indicated a journey over time from ‘intellectually’ understanding the process of building a compassionate mind to experiencing a more embodied sense of compassion that had significant impacts on their orientation to (and working with) the psychophysiological processes of bipolar disorder.

## Introduction

The bipolar cluster of disorders, which include bipolar I, bipolar II and cyclothymic disorders, are major, debilitating forms of mental health difficulties (Grande et al., 2016). The incidence for the narrow definitions is estimated at around 1–2.4% but becomes higher if one includes the cyclothymic disorders (Merikangas et al., 2011; Grande et al., 2016). Latalova et al. (2014) suggest that 25–50% will make at least one suicide attempt and 8–19% will eventually die by suicide. The risk of suicide increases in the presence of hopelessness, previous attempts, family history, poor social relationships, and substance abuse (Simpson and Jamison, 1999; Latalova et al., 2014). Bipolar disorders are affected by the quality of early and current family and social relationships (Greenberg et al., 2014). Early trauma is especially important (Aas et al., 2016). Trauma in younger patients with a diagnosis of bipolar disorder is associated with increased suicide risk, depression severity and poor functioning compared to non-traumatised patients (de Azambuja Farias et al., 2019). Bipolar disorders also have a range of detrimental effects on family, social and work relationships which further compromises the potential for helpful social relationships (Greenberg et al., 2014). Indeed, given the impact of social support and understanding for prognosis, a number of researchers have highlighted the importance of addressing styles of social relating for these individuals (Owen et al., 2017). People with a diagnosis of bipolar disorder also have elevated physical health risks. For example, Weiner et al. (2011) point out that people with this diagnosis may have nearly double the risk of cardiovascular disorders than the general population.

Although there are genetic and physiological processes underpinning vulnerability to this family of disorders (Gordovez and McMahon, 2020) these processes interact with trauma and a range of psychological and psychosocial factors (Aas et al., 2016). Hence, various psychosocial therapies have been developed as adjuncts to pharmacotherapy offering psychoeducation, cognitive and behavioural skills training (Vieta et al., 2018; Davenport et al., 2019) and psychosocial interventions (Swartz and Swanson, 2014; Miklowitz et al., 2021). In addition, psychotherapies are increasingly seeking to become more integrative, biopsychosocial (Gilbert, 1989/2016, 1995, 2013; Cozolino, 2017; Music, 2019; Siegel, 2019; Gilbert and Simos, 2022) and process-focused (Gilbert and Kirby, 2019; Watkins and Newbold, 2020). This is because of the growing scientific understanding of the complex co-regulating links between biological, psychological, and social processes in the areas of causation, maintenance, recovery, and change (Cozolino, 2017; Haslam et al., 2018; Porges and Dana, 2018; Kumsta, 2019; Schore, 2019). Dodd et al. (2019) have also addressed the important area of emotion regulation for people with these diagnoses and raised issues over the discrepancies between state and trait emotion regulation strategies. Recent NICE guidelines highlight the need to develop therapies that are specifically designed for people with a diagnosis of bipolar disorder (Jauhar et al., 2016). Hence, this study explores the modification of compassion focused therapy (CFT) for people with a diagnosis of bipolar disorder which seeks to address different aspects of functioning in the areas of motivation and emotion regulation, cognitive and physiological processes, and social relationships.

### An Evolved, Biopsychosocial Approach to Therapy

Compassion focused therapy is an evolution informed, biopsychosocial approach to mental health difficulties that draws on interventions from a range of different schools of therapy (Gilbert, 2000, 2010; Gilbert and Simos, 2022). One guiding framework for pursuing a biopsychosocial integration for psychotherapy is evolutionary functional analysis (EFA) (Gilbert, 1995, 1998, 2019, 2020a; Gilbert and Bailey, 2000; Nesse, 2019; Workman et al., 2020). EFA highlights that evolved motivation and emotional processes are underpinned by evolved, complex physiological *if* A *then* do B (stimulus response) algorithms which give rise to brain states (Gilbert, 1984, Gilbert, 1989/2016; Gilbert and Simos, 2022). For example, if a stimulus indicates threat, this triggers defensive behaviour. If a stimulus indicates sexual opportunity, then this generates sexual arousal and courting behaviour. The motive of caring has the algorithm of *if* stimulus indicates distress or need, *then* this activates behaviours to alleviate them. Clearly, this is just the basic process. In reality, according to the species, there will be multiple complexities such as higher cognitive processes such as mentalizing (Luyten et al., 2020). Nonetheless, CFT targets the specific activation and cultivation of compassion motivation as the framework for interventions because it evolved with neuro- and psychophysiological regulators (e.g. oxytocin and changes to the vagus nerve; Carter et al., 2017) that have profound effects on a range of physical and mental health parameters (Brown and Brown, 2015; Mayseless, 2016; Seppälä et al., 2017; Vrtička et al., 2017). These facilitate prosocial behaviours and the building of caring supportive relationships that have health and wellbeing benefits (Kirby and Gilbert, 2017; Seppälä et al., 2017; Gilbert and Simos, 2022). In addition, compassion has long been recognised as a means to address problematic mental states and suffering in self and others, with a range of interventions, such as mindfulness and compassion focusing (Dalai Lama, 1995; Ricard, 2015) that can be adapted and adopted into western psychotherapy (Germer and Siegel, 2012; Gilbert and Simos, 2022).

**FIGURE 1:**
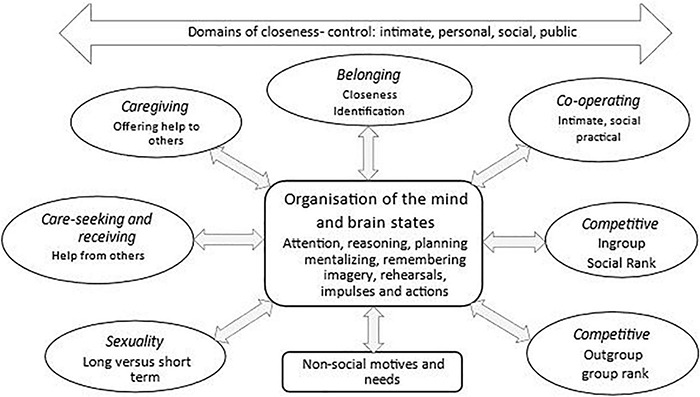
The organizing functions of motives and social mentalities. ©Paul Gilbert.

Compassion focused therapy distinguishes between non-social and social motives, and between different social motives (called social mentalities). Examples include: motives for care giving, seeking and responding to care, cooperating, competing and forming social ranks, group formation, and sexuality. Understanding these distinctions is important because evolved motives and their algorithms and multiple derivatives are not only linked to different physiological systems, but are the primary organising functions of the mind, with emotions and cognitive competencies being recruited to pursue the goals of a motive(s). Hence, as depicted in Figure 1, what we pay attention to, how we reason, the actions we plan and enact will be very different according to whether we are caring for somebody, seeking a sexual relationship, competing or arguing with them or seeking help from them. Importantly too, the physiological patterns and brain states will be quite different according to our motives. Differences in social motives are also impacted by the *domains of closeness*, the nature of the relationship such as how intimate or more distant they are in public to us. Important too is whether the relationship is sought out and wanted or whether it’s imposed by others, a voluntary or involuntary form of relating.

Compassion focused therapy suggests that social motives are different to non-social motives and are called social mentalities (Gilbert, 1989/2016). They create different types of social reciprocal roles, partly because social motives co-evolve to create dynamic reciprocal patterns of interactions. For example, infants could not evolve care seeking behaviours if ‘parents’ were not also evolving abilities to detect infant needs and distress and respond appropriately. Powerful, dynamic reciprocal patterns of interactions emerge as mother and infant come to co-regulate each other (Nguyen et al., 2020). Submissive behaviour and communications could not evolve unless individuals in dominant positions also evolved competencies to detect such signals and de-escalate conflict. Sexual behaviour is another obvious relational form that has to co-evolve in terms of mutual stimulation of sexual receptivity. Social mentalities require specialist processing systems to decode specific social signals in terms of their meaning, the motivation of the sender and the selection of appropriate responses for specific roles (e.g., caring, competing or sexual) (Gilbert, 1989/2016, 2005, 2019). Hence, we have different processing systems for co-creating (say) sexual relationships, caring relationships, dominant-subordinate relationships and ingroup-outgroup relationships. Individuals may be sensitive and competent in some role relationships but not all (Liotti and Gilbert, 2011). For example, some individuals may interpret a friendly signal as exploitive or dangerous; be inappropriately trusting or mistrusting; maybe caring or callous in the face of distress. CFT suggests many mental health problems are linked to problematic pursuits, interactions and conflicts between different social mentalities both between and within individuals. One common conflict is competing for social control, status and resources, in contrast to creating sharing, caring and supportive connections and relationships between self and others (Gilbert, 1989/2016, 2000, 2010, 2020a, 2020b, 2020c, 2021).

### The Problem of Competitiveness and Social Rank Evaluation in Bipolar Affective Disorder

A major social challenge for many species is how to secure resources when conspecifics are seeking (competing) to gain and control the same resources. Hence, a major evolutionary and social challenge was how to live in social groups where individuals would challenge each other. Price (1972) was one of the first to argue that going *up and down the ranks* had very different impacts on invigorating or deactivating behaviour, mood and physiology. In order to inhibit constant fighting, with the risk of injury, mechanisms for the judgement of social rank evolved (Price, 1972; Gardner, 1982; Price and Sloman, 1987; Gilbert, 1992; Sloman and Gilbert, 2000). Winning required different subsequent behaviours to losing. Winning triggers behavioural activation to take advantage of winning, elevated drive-linked positive affect and feeling energised, confident and explorative. In contrast, losing, and where there was a likelihood of losing again and/or where those more powerful would be vigilant and hostile to efforts to one’s ‘up rank bids’ for resource control, required a reassessment of one’s ability to challenge or defend against others (Sloman et al., 2011). In these contexts, there is a behavioural deactivation/demobilisation, turning off confident, explorative and resource seeking motivation, down-regulation of positive affect, awareness of inferior status/ability and vulnerability, with (often but not always) tendencies to be submissive, appeasing and socially anxious and/or to socially withdraw (see Gilbert, 2000, 2020b; Gilbert and Basran, 2019 for a recent review). Hollis and Kabbaj (2014) provide an extensive review of the physiological, psychological and social changes that occur in animals following defeats and how they closely map processes identified for certain depressions. Sloman (2000) labelled the defeat response an involuntary defeat strategy (IDS). In order to further research on the experiences of defeat, and internal and external entrapment, Gilbert and Allan (1998) developed a self-report measure for defeat and entrapment. Many studies across different client groups have now shown that these are different but interacting processes that are highly correlated with depression and suicidality (Gilbert et al., 2002; Taylor et al., 2011; Siddaway et al., 2015; Wetherall et al., 2019; Höller et al., 2020).

In regard to the role of competitive and dominant-subordinate behaviour in relationship to bipolar disorder, over 50 years ago, Janowsky et al. (1970) drew attention to the competitive and dominant type behaviours of (hypo)manic patients, including with their clinicians. Gardner (1982) further developed the idea that the phenomenology of bipolar disorders links to evolved processing systems that regulate competitive social rank behaviour. Just as in winning social contests and becoming dominant, animals can become energised in their resource seeking behaviour and sexuality, those losing and being socially defeated become socially withdrawn with changes in physical states (Gardner, 1982; Sloman et al., 2011). Wilson (1998) noted that the epidemiology for bipolar disorder was high, indicating positive selection for phenotypic variation in response to the challenges of resource competition in social hierarchical contexts. Johnson et al. (2012) also suggested that the striving for *social dominance* motivation system regulates mood, attention and energy, and underpins bipolar disorders. While people with unipolar depressions are often striving to avoid unwanted inferiority associated with shame and being rejected (Gilbert et al., 2007), those who shift into more (hypo)manic states can see themselves as superior and are striving to elevate their social control and social status (Johnson et al., 2005, 2012). When in elevated mood, people with a diagnosis of bipolar disorder tend to see themselves as superior and have a sense ‘specialness’ (Jamison, 2015). Gilbert et al. (2007) found that in a relatively stable group of people with this diagnosis, their mood variation was linked to elevated changes in social comparison where positive emotion was associated with feeling superior and more talented, competent and attractive than others, indicating that their positive emotion seems to be overly linked into rank evaluative systems. For non-bipolar people, however, elevated positive emotion such as winning a lottery is not necessarily associated with a change in social cognition such that one feels ‘superior, more talented or special’.

Further evidence that the competitive social mentality underpins bipolar disorder comes from Johnson and Carver (2006). They found that individuals vulnerable to bipolar disorder set high ambitious goals particularly for fame, wealth and political influence but less so for other prosocial goals. Similarly, Gruber and Johnson (2009) found that people with a diagnosis of bipolar disorder scored higher on interest in competitive goals, particularly of achievement, and less on compassion goals. In a recent review Ironside et al. (2020) note that patients with this diagnosis set extremely high goals for themselves and see achieving those goals as central to their self-worth. They are therefore highly sensitised to cues of reward and show elevated responses to laboratory manipulations involving success (Meyer et al., 2010; Pavlova et al., 2011), with less reactiveness to failures unless in a depressed phase (Wright et al., 2008). In a major review, Gruber (2011) noted that ‘recent research has suggested that bipolar disorder is associated with elevations in positive emotion specifically to reward and achievement-oriented emotions relative to pro social emotions’ (p. 359). In addition, when people move into hypomanic states, they can become impulsive with poor emotion regulation (Musket et al., 2021), grandiose, and if blocked, confrontative with authority (Janowsky et al., 1970). Hence, a number of researchers have highlighted the fact that the mechanisms regulating competitive behaviour, social rank and self-evaluation, are unstable in people with a diagnosis of bipolar disorder. This makes them vulnerable to extremes of ‘up and down rank brain state shifting’. The clinical question therefore is ‘is it possible to help clients understand this motivational system and how to cultivate a different motivational system which will introduce more psychophysiological stability?’ The candidate for this is the caring system (Gilbert, 1989/2016, 2020a; Cassidy and Shaver, 2016; Mayseless, 2016).

### The Evolution of Caring and Compassion

Caring and compassion motives evolved for very different reasons than those for conflict competition and have very different impacts on brain states and reciprocal interactions (Gilbert, 1989/2016, 2020b; Goetz et al., 2010; Mayseless, 2016). One of the primary functions of caring is to provide resources that support the survival of offspring into adulthood (Brown and Brown, 2015; Cassidy and Shaver, 2016; Music, 2017). The evolution of attachment behaviour brought with it competencies to be sensitive to the developmental needs of offspring, such as for feeding, thermoregulation and protection and provide for their psychophysiological maturation and development. Hence, the evolution of caring behaviour created new algorithms, rooted in particular psychophysiological systems, that attuned attention, social processing and action systems to the ‘role of caring’ (Bowlby, 1969; Gilbert, 1989/2016; Cassidy and Shaver, 2016; Mayseless, 2016). Among the most salient physiological systems are those related to oxytocin and endorphins (Depue and Morrone-Strupinsky, 2005; Carter, 2017; Carter et al., 2017; Siegel, 2020) changes to the parasympathetic nervous system, and in particular the myelination of the vagus nerve (Porges, 2017, 2021a,2021b) and a range of specific neurocircuits (Vrtička et al., 2017). Attachment theory and research have shown that over and above physical needs, the parent provides for a secure base and safe haven (Bowlby, 1969; Cassidy and Shaver, 2016). A secure base provides the context for learning, guidance and play and in humans, a sense of being loved and lovable, and that others are trustworthy and helpful. A safe haven soothes and calms distress or over excitement and provides inputs for physiological and emotional regulation. These inputs are fundamental to the development of the child’s emotional insights, tolerance and regulation and their basic orientation to the social world. In addition, support throughout life from partners and peers can play a fundamental role in helping people with a diagnosis of bipolar disorder cope with the disorder and being treatment adherent. Partner and peer social support have a particular impact on depressive symptoms and coping with depression (Greenberg et al., 2014).

Crucially, empathic caring stimulates important psychophysiological systems (as noted above) that enable the child to become caring of themselves and others and are linked to health and wellbeing (Brown and Brown, 2015; Mayseless, 2016; Music, 2017; Siegel, 2020; Ellis et al., 2021). Tragically when these inputs are not provided, and the child experiences non-empathic, hostile or neglectful ‘caring’ these impact their epigenetic profiles (Cowan et al., 2016), various neurocircuits (Lippard and Nemeroff, 2020), the basic psychophysiological core algorithms for enabling emotion and self-regulation, and the competencies to enter social life socially confident (Music, 2017; Siegel, 2020). They are more likely to become over reliant on threat-based, self-focused competitive strategies to get through life (Gilbert, 1989/2016, 2020c; Gilbert and Simos, 2022). When children are forced into being self-protective, they can take one of two social rank strategies. One is to be very vigilant of social risk and adopt appeasing, submissive lifestyles (Gilbert, 2020b). The other is to be more resource, achievement and power seeking, what Johnson et al. (2011) called social dominance seeking. There is growing evidence that these social rank processes are very important regulators in mood disorders (Sloman and Gilbert, 2000; Gilbert et al., 2002; Johnson et al., 2011; Taylor et al., 2011; Hollis and Kabbaj, 2014; Wetherall et al., 2019). They operate along multiple dimensions such as superior-inferior, entitled-undeserving, submissive-aggressive, winner-loser/defeated, competent-incompetent, self-blame vs. other-blame, wanted-unwanted, shame prone vs. shameless, self-critical vs. other-critical (Gilbert, 1992, 2020a). In addition, self-criticism (a target for CFT and linked to the competitive social mentality) in contrast to self-compassion and self-reassurance have been shown to stimulate different neurophysiological pathways (Longe et al., 2010; Kim et al., 2020).

### The Therapeutic Benefits of Motivation Switching

**FIGURE 2:**
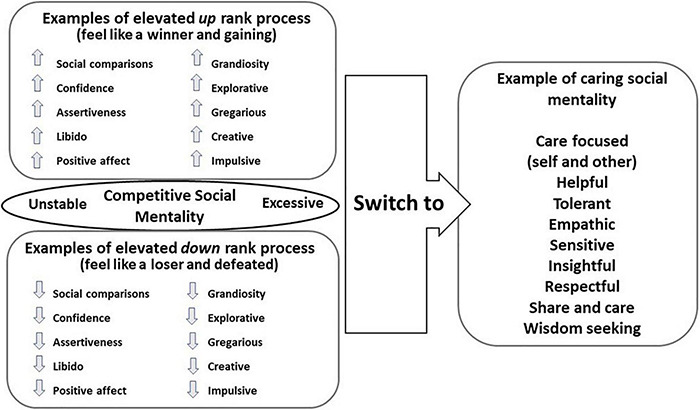
An overview of the instability of the process underpinning social ranking and the movement towards caring motivational process. ©Paul Gilbert.

As noted, there is general agreement that people with a diagnosis of bipolar disorder are characterized by swings between excessive positive mood and low mood associated with invigorated and demobilised behaviour and positive and negative self-evaluation (Power, 2005). Taking an evolutionary approach that seeks to identify the natural regulators of mood states, CFT follows the view that this instability is the result of problematic regulation of strategies and algorithms that regulate competitive behaviour in terms of navigating social challenges and social control (e.g., resource control and status). This algorithm can become internally regulated via biological oscillations, e.g., chronobiological rhythms that contribute to unstable and excessive oscillations within it (Gonzalez et al., 2019). Indeed, many models of bipolar disorder have highlighted the fact that individuals can switch into feeling dominant, with expansive feelings of confidence but also experience dramatic shifts into loss of energy, confidence, feeling defeated, inferior, worthless and incompetent. It is seeking to understand the functions of these changes in the context of a particular motivational process that can provide clues to the disorder. This is depicted in Figure 2. In essence then, for people with a diagnosis of bipolar disorder the evolved motivation and processing systems for the regulation of conflict and resource acquisition within social ranks, is unstable and vulnerable to excessive switches of engagement and disengagement of resource seeking behaviour. Although these are presented as if they are unidimensional, that is unlikely because the physiology of dominance is not the opposite of the physiology of subordinate defeat states. This allows for mixed states.

One way of working with people with this condition is to try to stabilise the rank system. However, CFT suggests that the attachment and caring systems evolved as psychophysiological, emotion and behavioural regulators and provide the foundation for well-being and health (Brown and Brown, 2015). Hence, therapies can seek to switch individuals out of defensive or excessive competitive motives into compassionate and caring ones. As noted above one reason for doing this is that it will shift not only a range of psychological processes but also physiological and social ones too. This shifting process is also depicted in Figure 2.

Any one motivation can have many different textures. For example, the way people with a diagnosis of bipolar disorder seek and pursue social dominance can be very different from how psychopaths or narcissists do. People with a diagnosis of bipolar disorder lack the callousness, aggressiveness and hostility of those with psychopathic traits. In some ways psychopathic strategies look ‘more fitted’ to earlier primate and more hostile hierarchical contexts, whereas bipolar forms of competition maybe more fitted to hunter gatherer hierarchies of competing for attractiveness and demonstrations of talent (Gilbert and Simos, 2022). What they have in common is how they socially compare themselves with others and the drive for status and resource control (Tharp et al., 2021). Similarly, people can be compassionate in different ways. For example, risking one’s life to save others as a firefighter or a person working on a COVID-19 ward might not necessarily mean they are most empathic parent. Different roles require slightly different empathic competencies (Liotti and Gilbert, 2011). These variations are because brain states are complex interacting patterns of motives and emotions linked to life history and phenotypic maturation. In addition, people can behave kindly and compassionately for many reasons, including those for being liked (Böckler et al., 2016). One form can be submissive compassion (Catarino et al., 2014). Nonetheless, CFT seeks to help clients to become motivationally aware and practise cultivating compassion because of its biopsychosocial effects (Weng et al., 2018; Singer and Engert, 2019; Kim et al., 2020). Consequently, it uses a range of multi-modal interventions that address the four functions of mind, namely: motives, emotions, cognitive competencies, and behavioural enactments (Gilbert and Simos, 2022). There is increasing evidence that specific compassion practises that target these functions can have specific neuro and psychophysiological effects (Weng et al., 2018; Petrocchi and Cheli, 2019; Singer and Engert, 2019; Porges, 2021a,b). Especially important is to help clients develop an attachment-like inner set of competencies that provide for secure base and safe haven functions in how they relate to themselves (Gilbert, 2020b).

### Compassion Focused Therapy

Compassion focused therapy defines compassion in terms of its evolved stimulus-response algorithm which is to be *sensitive to (stimuli of) suffering in self and others with a (response of) commitment to try to alleviate and prevent it* (Gilbert, 2020a). At times to be sensitive and engaged with suffering requires considerable courage and that includes with one owns distress. To take appropriate action can also require courage but in addition it requires wisdom. Courage without appropriate wisdom can be reckless or harmful and wisdom without courage can be ineffective. Clients are guided through this definition and to see that the core of compassion is to acquire the *courage* and *wisdom* to engage with suffering and difficulties and the *courage* and *wisdom* to work out, and practise what is helpful and to engage in appropriate action. There are six processes that support *engagement* with difficulties and distressed mind states. These include: to be motivated to develop compassion, becoming mindful and sensitive to emotional states, being sympathetically in tune with states of suffering and difficulty, being able to tolerate such states, being able to empathically understand and mentalize one’s mind and being open rather than condemning or pushing away what arises in one’s mind with changes of brain state. There are six suggested processes for *taking action* to prevent and alleviate suffering. These include: learning to remember to bring to one’s attention to what is likely to be helpful, using imaginary scenarios of what can be helpful, using mindfulness and compassionate reasoning, engaging in whatever appropriate helpful behaviour for the specific situation is required, using ‘body grounding’ when helpful, and tolerating as well as working with the emotions that can arise from taking helpful action. The same competencies are used when being compassionate to others.

These are contextualised in helping clients understand the nature of our evolved brains and how tricky they can be because they have been built by genes as vehicles to carry them into the subsequent generations (wisdom). We experience ourselves through the textures of motives and emotions made possible by gene-built and socially-shaped brains. In other words, our brains and minds were built for us, not by us. Clients begin to recognise that much of what is happening within them is not their fault but is part of activated, evolved motivational and emotion systems. These insights help them to then stand back, develop mindful observation skills and recognise that, rather than identify with any particular state of mind, they can begin to work with it (Germer and Siegel, 2012; Gilbert and Choden, 2013). This also involves mentalizing self and others (Luyten et al., 2020). Key to this process is to also help clients identify and work with hostile forms of self-criticism and unhelpful behaviors. Once people are aware and attentive to the evolved nature of mind, they can then practice switching to a compassionate focus and use various combinations of the twelve competencies outlined above. Hence, for any state of mind people are encouraged to consider ‘what would be helpful in contrast to harmful’.

Compassion focused therapy distinguishes compassion from kindness and other *ways of being* compassionate (Gilbert et al., 2019). As noted recently, in an important paper called *Compassion is not a Benzo*, Di Bello et al. (2021) highlight the fact that training in compassion does not mean that for any specific compassionate act people will be in a ‘calm mind’. For example, a firefighter or emergency COVID-19 clinician might not have a calm mind, but they are able to tolerate anxieties or other dysregulating emotions *to maintain focus on intention*. Having the physiological and psychological skills to hold intention is important and can be compared to physical training. Training exercises for compassion can be compared with getting fit. Getting fit does not mean one is in a state of activation all the time, but it means that when we need to run or engage in hard physical activity, we are able to do so, recover quickly and not collapse out of breath, unable to complete a task.

Although there is now clear research showing the beneficial effects of stimulating the psychophysiological mechanisms underpinning compassion, one of the challenges for therapy is that people carry many fears, blocks and resistances (FBRs) to it (Gilbert et al., 2011; Lawrence and Lee, 2014; Kirby et al., 2019). Among the fears are that compassion will make one weak or to lose ambition. People can also feel they do not deserve compassion (Pauley and McPherson, 2010). Another major problem is that stimulating compassion activates the attachment and care motivational systems. Traumas coded within those systems, such as neglect or abuse can become re-activated. Hence, rather than feeling connected, safe and relieved clients can begin to re-experience trauma associated with early attachment relationships (Gilbert and Simos, 2022). There is now considerable evidence that fears of compassion are linked to many mental health and anti-social behavioural problems (Kirby et al., 2019). Hence, these are core focuses of CFT (Gilbert and Simos, 2022).

Compassion focused therapy has proved successful for a range of *trans*-diagnostic and complex mental health difficulties (Basran et al., 2022) including those suffering with: psychosis and voice-hearing (Mayhew and Gilbert, 2008; Braehler et al., 2013; Heriot-Maitland et al., 2014), recovering from psychosis in a high-security setting (Laithwaite et al., 2009); personality disorders (Gilbert and Procter, 2006; Lucre and Corten, 2012) routine outpatients (McManus et al., 2018), community outpatient groups that included participants with a diagnosis of bipolar disorder (Judge et al., 2012), survivors of domestic and gender abuse (Naismith et al., 2020). Brown et al. (2020) found that elements of compassionate mind training, in particular developing a compassionate image (coach) increased compassion for others and reduced paranoia in individuals with elevated paranoia scores. Forkert et al. (2022) developed a four-session compassion imagery intervention for clients with persecutory delusions. They found that this compassion intervention was feasible, acceptable, and based on qualitative analyses, was experienced as being helpful. This included participants ability to manage their difficulties and, in some cases, to feel safer and more self-accepting. Gilbert and Procter (2006) developed a module-based compassionate group therapy and mind training approach for day hospital patients. Their feedback, along with work with a number of groups and colleagues (see Gilbert and Simos, 2022 for examples) over the years have generated developments in group based CFT guidance and now there is a manual in preparation for publication (Gilbert et al., 2016–2022). Arnold et al. (2021) and Cattani et al. (2021) have developed a student-focused group CFT manual from our prepublication manual. Lowens (2010) was the first to develop an outline of CFT for people with a diagnosis of bipolar disorder. He highlighted the need to pay attention to clients’ tendencies for high self-criticism, difficulties with emotional regulation and engagement with compassion. Although not a research study, he articulated a number of areas where cultivating compassion was a challenge but when achieved was particularly helpful. Although some of the authors of this paper have experience of working with people with bipolar disorder, this is one of the first studies to explore group based CFT on client experience and change processes with participants with this diagnosis attending a support service.

### Core Themes of Change

**FIGURE 3:**
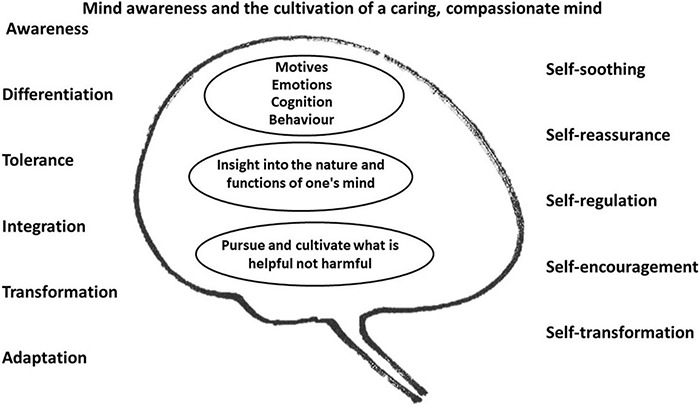
Core processes of change. ©Paul Gilbert.

Many psychotherapies seek to help clients with common processes that include: *Mind awareness*, *differentiation, tolerance, integration and transformation* which increase people’s biopsychosocial abilities to *adapt* to changing internal and external events (Gilbert and Kirby, 2019; Gilbert and Simos, 2022). All these facilitate contextually appropriate psychophysiological (wise) flexibility. In addition they build on each other. For example, awareness facilitates differentiation (e.g., of motives, desires, emotions or beliefs) and differentiation increases awareness. Differentiation supports tolerance and tolerance supports openness to explore and differentiate processes within one’s inner mental life. CFT addresses these issues through evolution informed psychoeducation, integrating a range of standard evidence-based interventions such as body focused practices, mindfulness, cognitive reappraisal, visualisation/imagery, empathy training, validation, developing emotional awareness and tolerance and behaviour exposure practises and rehearsals. An overview is presented in Figure 3. These processes in turn develop people’s ability to be self-soothing, reassuring and encouraging and can transform people’s sense of an identity.

What is unique to CFT is how basic interventions are contextualised in psycho-education about why the human brain is tricky and can create these distressing states of mind. Also is the importance of recruiting the psychophysiological properties of the evolved caring social motive system (Vrtička et al., 2017; Singer and Engert, 2019; Gilbert and Simos, 2022). For example, cognitive reappraisal can be generated following a compassionate mind induction that involves particular breathing practises, body grounding and utilising the wisdom and intentionality of compassion. There is increasing evidence that specific practises and intention focusing can have major neurophysiological (Singer and Engert, 2019; Ashar et al., 2021) and physiological effects (Matos et al., 2018). Hence, one of the aims of a compassionate mind priming is to involve activation of the vagus nerve, oxytocin and various neurocircuits (particularly in the frontal cortex) known to be linked to compassion and to have threat regulating properties (Weng et al., 2018; Ashar et al., 2021; Matos et al., 2021).

### Heart Rate Variability Profiles in Patients With a Diagnosis of Bipolar Disorder

As indicated, CFT is a biopsychosocial approach to therapy. In regard to the ‘bio’ aspects and related to neuroplasticity, compassion training can change a range of neurocircuits (Singer and Engert, 2019). Kim et al. (2020) highlighted the different neural signatures when people were being either self-reassuring or self-critical to a disappointment, rejection or a failure. They showed that the neural networks associated with threat processing are reduced with compassionate mind training (CMT). In addition, CMT significantly improved heart rate variability (HRV). HRV, especially the regulating functions of the vagus nerve, are linked to a range of psychological and health processes, prosociality (Porges, 2007, 2021a,2021b; Keltner et al., 2014; Kogan et al., 2014) and a sense of social safeness (Kelly et al., 2012). Social safeness may act as an emotion regulation process in its own right, linking a sense of caring social connectedness to such physiological benefits (Armstrong et al., 2021). There is evidence that people with a diagnosis of bipolar disorder lack balance between sympathetic and parasympathetic activation (sympathovagal unbalance), with resulting reduced heart rate variability (Levy, 2014; Gruber et al., 2015; Wang et al., 2016). In comparison with healthy controls, patients with this diagnosis tend to show lower resting HRV (Chang et al., 2014; Faurholt-Jepsen et al., 2017, for review; Henry et al., 2010; Quintana et al., 2016). Stimulating caring and compassion motives increases parasympathetic activity as measured through HRV (Rockliff et al., 2008; Matos et al., 2017; Petrocchi and Cheli, 2019; Kim et al., 2020; Steffen et al., 2021). Indeed, HRV is a recommended outcome measure for assessing CFT (Kirby et al., 2017) and a recent meta-analysis confirmed a significant association between compassion and HRV with a medium effect size (Di Bello et al., 2020).

Whilst there has been anecdotal evidence that CFT can be helpful to patients with a diagnosis of bipolar disorder (Lowens, 2010) and that CFT can increase HRV in ‘healthy’ populations (Rockliff et al., 2008; Matos et al., 2017; Kim et al., 2020) and depressed students (Steffen et al., 2021), to date, no studies have assessed how people with bipolar disorders will experience a group based CFT, whether they will find it helpful and whether CFT can alter their HRV. To explore the latter, we first sought to explore if CFT impacts on baseline patterns of HRV. Second, we sought to explore if CFT can impact on HRV to specific evolutionary important themes. Gruber (personal communication) suggested that it can be revealing to explore how clients respond to certain provocations linked to their condition. Hence, we designed a competitive scenario and a social connectedness scenario. In the competitive scenario clients were invited to imagine winning and losing a competition. In the connectedness scenario participants were invited to imagine feeling socially connected and belonging and then loneliness. In general then, we sought to explore how people with this diagnosis will experience the psycho-education on evolution in relationship to their disorder, motivational shifting from rank focused to care focused processing and the practices to activate and cultivate compassion motive systems (Figure 2).

### Aims

The aim of the present study was to examine the experience and feasibility of a 12 module CFT group tailored for individuals with a diagnosis of bipolar disorder. The participants were clinically, and personally regarded themselves as relatively stable, not currently suffering from any major depression or hypomania episodes. They were invited as collaborators in this research project to explore the feasibility of CFT and to help modify current compassion focused interventions for bipolar disorder. We hypothesized that if participants were able to understand the nature of our ‘tricky brain’ and how motives organise the mind, and therefore the value of switching from a competitive focus to a compassion focus motivation orientation, this would have significant impact on coping with their bipolar disorder. In particular, there would be reductions in self-criticism and increases in self-compassion and openness to compassion. We studied changes in self-report measures and HRV at the beginning and end of the therapy, as well as changes in HRV related to specific scenario activations. In addition, we qualitatively explored and analysed participants’ detailed insights and reflections from three focus groups provided at different timepoints.

## Materials and Methods

### Design

The study employed a repeated measures within-subjects design using self-report and HRV measures, as well as qualitative focus groups and therapist observations. The initial design proposed to deliver 12 modules of group CFT to explore its feasibility. As participants were invited as collaborators as part of the research study, we kept very close attention to their experience and their suggestions. They became very engaged with the therapy, and wanted to spend more time on each of the themes and content of the module than had been anticipated. Consequently after 12 sessions, a number of modules had not been completed and both participants and the therapists were very keen to see if we could extend the number of sessions so that the materials planned could be covered. Participants felt that 12 sessions were far too short and as the qualitative data will reveal they felt they had ‘only just got started’. So, with agreement from the participants and ethics, it was extended from 12 to 25 sessions (additional 13 sessions). The sessions were delivered once a week (where possible) for a duration of just over 47 weeks. There was a break of 2 weeks during the first 12 sessions due to Easter holidays and other contingencies and a 20-week gap between the two parts due to seeking permission for the extension from the ethics committee.

Self-report and HRV measures were collected at baseline; at the end of the first set of 12 sessions of group CFT; and after the 20-week break (and before the commencement of the second set of sessions); and finally at the end of the second set of sessions. Focus groups were conducted at all three time points after baseline: after the first 12 sessions, just before the start and then at the end of the second set of sessions. A further 1 year follow up was planned, unfortunately due to the COVID-19 pandemic, this was not possible.

### Participant Engagement Process

One of the therapists, who is employed by a United Kingdom specialist Bipolar Service and interested in working with CFT, advised clients of the potential for research in a compassion focused approach for those with a diagnosis of bipolar disorder and explored potential interest. Subsequently, PG who has run a number of CFT groups for complex cases, visited the bipolar service and offered a 1.5-h introduction on the evolutionary model of bipolar disorder (as related to social rank instabilities) and the nature of compassion. They then explored if the participants would be interested in taking part in a study exploring the impact of cultivating compassion. Some participants were enthusiastic and acknowledged that the idea of ‘instability of social rank mechanisms’ as rooted in evolutionary mechanisms that were not their fault was a novel and helpful way to think about their mood difficulties. They were intrigued by the idea that they could ‘maybe’ learn to explore and use compassion motives to be helpful. Approximately 6 months later, they along with other clients from the service were invited to participate in the study by their clinician who also took informed consent.

Inclusion criteria were that participants had a clinical diagnosis of, and had been treated for, a bipolar affective disorder and were relatively mood stable. The exclusion criteria were (i) being severely depressed or hypomanic, (ii) having other major mental health issues such as drug or alcohol use problems or organic complications that could interfere with the reliability of the study, and (iii) clinical or self-assessment as a suicide risk. Ethical approval for the study was obtained from London Central NRES Committee (REC ref 18/LO/1234 IRAS 248283).

Initially, 13 participants were recruited into the study, three of whom decided to withdraw before the group commenced as they were unable to attend all the sessions. The final group consisted of 10 participants, 6 women and 4 men aged 31–60 years old with a diagnosis of bipolar disorder. All participants had previously attended the Bipolar Service where they received an intervention designed for those with a bipolar or cyclothymia diagnosis called Mood on Track. This draws heavily on a number of therapies including CBT, mindfulness and family therapy. During the course of the study, two participants withdrew because of work-related reasons, and two withdrew because they were offered additional support for their needs at the time which may have influenced the change process. Therefore four women and two men (aged 31–59) completed the full 25 sessions of CFT, attending an average of 82% of the sessions. All participants were taking medication, the most common of which was lithium (*n* = 4, associated with minimal effects on HRV; Bassett, 2016), followed by anti-psychotics (*n* = 3, associated with reduced HRV; Huang et al., 2016), *anti-depressants* (*n* = 2, associated with reduced HRV; O’Regan et al., 2015), anti-convulsant medication (*n* = 1), blood pressure medication (*n* = 1), thyroid medication, (*n* = 1), pain (*n* = 1) and sleep medication (*n* = 1).

**FIGURE 4:**
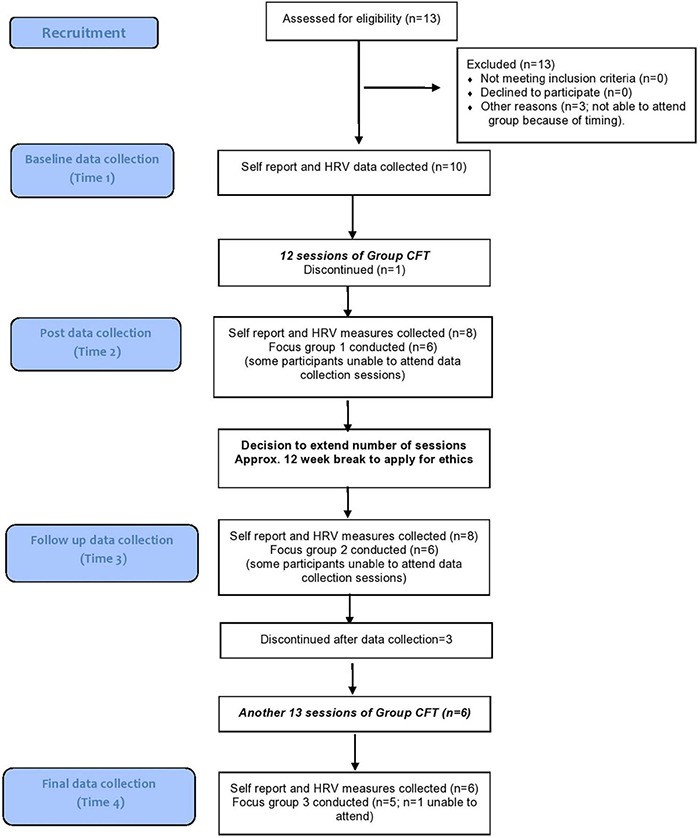
Participant flow.

For the full participant flow, refer to Figure 4.

### Intervention

Compassion focused therapy was delivered by two qualified clinical psychologists (AR and AH). AR has extensive experience with CFT, and AH has attended CFT training and has extensive experience delivering psychological therapies for people with a diagnosis of bipolar affective disorder. They were supervised regularly by the founder of CFT (Gilbert, 2000, 2009, 2020a). Another author (KL) facilitated the process of the project within the Trust.

As this study was a proof of principle, the initial plan was to deliver a standard 12 module program over 12 weeks. However, as participants became very engaged with the therapy, with desires to discuss it with the therapists and each other in detail, it became clear that the time necessary for working with this group was insufficient. Hence, the therapists and participants sought to extend the intervention. The ethics committee was contacted at the end of the initial 12 sessions to request an extension. This process took over 20 weeks during which time participants did not receive any active therapy but were able to practise the CFT exercises they learned during the first 12 sessions. Consequently, the intervention consisted of the original 12 sessions over 12 weeks (14 weeks in total when 2-week break due to holidays considered), a 20-week rest period and then another 13 sessions over 13 weeks. Therefore, the 12-module content was covered in 25 sessions over a total duration of 47 weeks when holidays and break (for ethics) considered.

Group therapy was delivered once a week (where possible) covering material from a standardised manual for CFT using a modular approach derived from a manual in preparation (Gilbert et al., 2016–2022). Generally, the modules focus on specific themes and can take more than one session. For example, the self-criticism module was covered over three sessions. Additional sessions also focused on therapeutic processes such as when participants disclosed traumatic experiences or identified particular fears, blocks or resistances to compassion. Key to group CFT is the use of flip charts to regularly write up participant ideas, feedback and discussion. Therapists use participant feedback to draw diagrams of processes for reflection, and practises such as mindfulness and soothing rhythm breathing and creating compassionate images can be recorded, and copies given to clients. Summaries of sessions can be written up and given to participants with key points. The standard 12 modules are covered in Table 1.

**TABLE 1:** Module outline.

Module	Theme	Summary
1	Introductions, setting working agreements, and exploring basic concepts of compassion	It starts with introductions, discussing the procedures for safe engagement such as confidentiality and respectfulness, and introduces the evolution informed concept of compassion with working definitions on how compassion affects the body and the mind.
2	Psychoeducation and the evolutionary model	This module explores the basic psychoeducation of the evolutionary model including concepts of ‘tricky brain’, motivational system conflict and the functional analysis of emotions (threat, drive, and soothing).
3	The nature of caring behaviour, secure base and safe haven	This module involves deepening the understanding of compassion and linking it to the functions of caring (as identified in attachment theory) as a secure base and safe haven. Participants are guided into how to create these in the group and how to create them within oneself. Therapists help participants distinguish between safeness and safety and how to use imagery to create a sense of safeness.
4	Attention and mindfulness	This module introduces participants to the ‘power of attention’ and its link to psychophysiological functioning. Participants are then introduced to the nature of mindfulness and some basic mindfulness practices. An important aspect is to learn to identify unhelpful loops of thinking and how to mindfully switch to compassion focusing.
5	Building the compassion mind and self	This module guides participants on how to build a compassionate mind and body using a range of compassion focus practices including breathing, posture, thinking and imagery and compassionate writing to and about oneself.
6	Openness to compassion and compassionate image	This module guides participants to the process of receiving compassion. One vehicle for this is via developing their compassionate image of another mind being compassionate to them, and learning how to create compassion-focused dialogues to support internalising a secure base and safe haven. In addition, participants explore gratitude memories and how they are open to giving and receiving of compassion to and from others.
7	Multiple selves and mixed brain states	This module introduces how to explore, become aware of and work with, our multiple and often conflicting motives and emotions. Participants are guided through specific CFT practices which help clients identify the complex interacting emotions. These support processes of awareness, differentiation, tolerance, integration, and transformation (see Figure 3).
8	Self-criticism	This module helps participants explore the nature of harmful self-criticism. They are guided through a functional analysis of shame-based self-criticism and self-attacking and how to contrast that with, and develop, compassionate self-correction, reassurance and encouragement as associated with secure base and safe haven functioning.
9	Shame and guilt	This module explores the nature and differences between shame and guilt and how to work with both using the compassion skills and insights provided in the therapy so far.
10	Assertive apologies and forgiveness	This module guides participants through the three domains of assertiveness (e.g, resisting, promoting and accepting praise), apologies and forgiveness in working with relational conflict. Attention is also given to developing caring, valuing and sharing relating styles and using the group as examples.
11 and 12	Reflections and progress	This module invites reflections on the therapy, individual progress and difficulties and offers help with practices. In addition, an important process is coming together at the end of the sessions, issues of separation and ‘saying goodbye’.

A brief description of the module recommendations for compassion focused therapy are given in Supplementary Appendix 1. These are derived from an ongoing manual development (Gilbert et al., 2016–2022). Group psychotherapist Burlingame et al. (2018) attended training in CFT in the United Kingdom and utilised the manual noted above for working with university students (Arnold et al., 2021; Cattani et al., 2021). They have proceeded with research with good evidence for effectiveness (Fox et al., 2020; Steffen et al., 2021).

In this study the therapists followed the manual with some adaptations partly due to the fact that these clients, as part of the Bipolar Service, had already participated in a programme which draws heavily on CBT, family-focused therapy and mindfulness-based interventions, called Mood on Track. This programme includes 11 group sessions and a number of individual sessions alongside an ongoing monthly support group. As such, initially, the mindfulness module was touched on briefly and later expanded on to introduce opportunities to practise mindfulness and to facilitate a understanding of how mindfulness supports compassion. Moreover, some modules took longer than anticipated, therefore due to time constraints the module on assertiveness, apology and forgiveness was not covered.

### Measures

#### Feasibility

As this was the first study to explore CFT group therapy for people with a diagnosis of bipolar disorder, we will identify the core themes recommended by Bowen et al. (2009). We are grateful to one of the reviewers for highlighting this approach. These include:

*Demand* arises from the epidemiology of the difficulty and need for, and availability of interventions.
*Acceptability* relates to how participants experience and react to an intervention.
*Implementation* addresses the degree to which an intervention can be fully implemented as planned and proposed.
*Practicality* addresses the issues of time commitment and resource availability.
*Adaptation* is concerned with any modifications as may be needed to accommodate different formats, media or populations.
*Integration* relates to the process of integrating the interventions into systems of care delivery.
*Limited-efficacy testing* relates to the evidence for preliminary effectiveness.

### Self-Report Measures

The following self-report measures were completed:

#### Depression, Anxiety and Stress Scale

The Depression, Anxiety and Stress Scale (DASS) (Lovibond and Lovibond, 1995) measures symptoms of depression ‘I felt that life was meaningless’, anxiety ‘I felt I was close to panic’ and, stress ‘I found it difficult to relax’. For this study, we have opted to use the short form of the original 42-item DASS. The 21-item short form has seven items from each of the original three subscales. Participants respond to the items on a four-point Likert scale ranging from 0 (did not apply to me at all) to 3 (applied to me very much, or most of the time). This scale has high internal consistency, with Cronbach alphas of 0.94 for depression, 0.87 for anxiety, and 0.91 for stress (Antony et al., 1998).

#### The Hospital Anxiety and Depression Scale

The Hospital Anxiety and Depression Scale (HADS) was developed by Zigmond and Snaith (1983) to provide a measure of severity of depression and anxiety in general medical environments. It consists of 14 items, seven of which measure depression, and the other seven anxiety, scored on a four-point Likert scale ranging from 0 to 3. The scale has high internal consistency for both anxiety (0.92) and depression (0.88) subscales (Djukanovic et al., 2017).

#### The Experiences Questionnaire

The Decentering subscale of the Experiences Questionnaire (EQ; Fresco et al., 2007) is an 11-item self-report instrument that assesses the construct of decentering, an ability to observe one’s thoughts and feelings as temporary, objective events in the mind, as opposed to reflections of the self that are necessarily true. Sample items include “I am better able to accept myself as I am” and “I can observe unpleasant feelings without being drawn into them”. The Decentering subscale has good internal consistency (0.83; Fresco et al., 2007).

#### Positive Affect and Negative Affect Scale

The Positive Affect and Negative Affect Scale (PANAS) (Watson et al., 1988) consists of two 10-item mood scales and was developed to provide brief measures of positive and negative affect. The items were derived from a principal components analysis of Zevon and Tellegen’s (1982) mood checklist. Participants are asked to rate the extent to which they have experienced each particular emotion within a specified time period, with reference to a five-point scale. A number of different timeframes have been used with the PANAS and we adopted ‘during the past week’ for this study. The scale has good internal for both factors, positive affect (0.90) and negative affect (0.91) (Serafini et al., 2016).

#### Three Types of Positive Affect Scale

Gilbert et al. (2008) developed this scale to measure the degree to which people experience different positive emotions. Participants are asked to rate 18 ‘feeling’ words on a five-point scale to indicate how characteristic it is of them (0 = ‘not characteristic of me’ to 4 = ‘very characteristic of me’). Factor analysis revealed three factors or subscales, these are: Activated Positive Affect (e.g., “excited”, “dynamic”, “active”); Relaxed Positive Affect (e.g., “relaxed”, “calm”, “peaceful”) and Safeness/Contentment Positive Affect (e.g., “safe”, “secure”, “warm”). The scale showed good psychometric properties with Cronbach alphas of 0.83 for Activating Positive Affect and Relaxed Positive Affect, and 0.73 for Safeness/Contentment Positive Affect (Gilbert et al., 2008).

#### Forms of Self-Criticism/Self-Reassuring Scale

The Forms of Self-Criticism/Self-Reassuring Scale (Gilbert et al., 2004) consists of 22-items and assesses participant’s self-critical thoughts and feelings about themselves during a perceived failure. Two subscales measure forms of self-criticising (inadequate self and hated self) and one subscale measures tendencies to be reassuring to the self (reassured self). Participants are asked to rate how they typically think and react when things go wrong for them and to respond on a five-point Likert scale (0–4). The scale has good reliability with Cronbach’s alphas of 0.90 for inadequate self, 0.86 for hated self, and 0.86 for reassured self (Gilbert et al., 2004).

#### Social Comparison

This scale was developed by Allan and Gilbert (1995) and consists of 11 bipolar constructs regarding rank and relationships with others in society. Each item is rated in a 10-point Likert scale. Low scores indicate to feelings of inferiority and general low rank self-perceptions. The Cronbach alpha was 0.91 in students and 0.93 in patients (Allan and Gilbert, 1995).

#### Social Safeness and Pleasure Scale

The Social Safeness and Pleasure Scale (SSPS) (Gilbert et al., 2009) was developed to assess the extent to which individuals feel a sense of warmth, acceptance, and connectedness in their social world. Participants rate their agreement with 11 statements using a Likert scale from 1 (“almost never”) to 5 (“almost all the time”). Previous research has found that this scale demonstrates good internal consistency (0.96) (Kelly and Dupasquier, 2016).

#### Compassion Engagement and Action Scale

The Compassionate Engagement and Action Scales (Gilbert et al., 2017) are three scales which measure self-compassion (“I am motivated to engage and work with my distress when it arises”), the ability to be compassionate to distressed others (“I am motivated to engage and work with other peoples’ distress when it arises”) and the ability to receive compassion from key persons in the respondent’s life (“Other people are actively motivated to engage and work with my distress when it arises”). In the first section of each scale, six items are formulated to reflect the six compassion attributes in the CFT model: sensitivity to suffering, sympathy, non-judgemental, empathy, distress tolerance and care for wellbeing. These sections also include two reversed filler items. The second section of the scale has four more items which reflect specific compassionate actions to deal with distress and an extra reversed filler item. Filler items are *not* included in the scoring. Participants are asked to rate each statement according to how frequently it occurs on a scale of 1 to 10 (1 = Never; 10 = Always). In their original study, the CEAS showed good internal consistencies and temporal reliability (Gilbert et al., 2017).

### Heart Rate Variability Measurement

For controlled collection of heart rate variability (HRV) data, participants were asked to refrain from: (a) eating; (b) drinking tea or coffee; and (c) strenuous exercise, 2 h preceding the scheduled appointment. They were asked to: (a) follow a normal sleep routine the day before, recording sleep and wake times; (b) avoid intense physical training the day before, and (c) avoid alcohol for 24 h (Laborde et al., 2017). Upon arrival at the Bipolar Service, participants were welcomed by a clinician (unrelated to the delivery of their CFT course) and were seated whilst the clinician assisted them in attaching disposable electrodes to their wrist and ankle to measure their HRV via Biopack software. Participants were asked to sit as still as possible with both feet flat on the floor and their palms upwards on their thighs whilst they were led through two rank and two attachment-based imagery scenarios with neutral scenarios in between. Scenarios were presented using a PowerPoint presentation to aid with standardisation and timing of the scenarios, which lasted 3 min each with the neutral scenarios lasting 2 min each.

Before the first scenario, a short set of instructions were given to introduce the imagery task: *‘When imagining the situations, they don’t have to be actual memories. We would like you to use your imagination. You do not need to try to create vivid pictures in your mind when you are creating your imagined situations. It’s more important that you try to put yourself in a state of mind of being in that situation and the kind of feelings and thoughts that might arise for you. You may find that your mind wanders and you might not be able to keep it on task for very long. This is because our minds often wander and think about a range of things at the same time. Do not worry about this, just bring it back to your focus’*. A presentation then guided participants to imagine two competitive scenarios (winning and losing) and two social connection scenarios (feeling connected to others and feeling lonely). For example, in the losing scenario participants were instructed to: *‘Try to imagine yourself in a situation where you feel defeated, and other people seem to have done better than you. This could be losing a competition or being rejected at a job interview’.* In the social disconnection scenario participants were instructed to: *‘Try to imagine yourself in a situation where you feel lonely and isolated, and less connected to other people. This could be feeling different to other people and not being part of the group’.* In an attempt to bring participants back to a neutral state, a scenario of imagining walking through a bookshop was used between each scenario. The sequence of scenarios was defeat, winning, lonely and connected, with neutral tasks in-between.

### Focus Group

There is increasing evidence that people diagnosed with depressions may have very different subjective experiences and symptom profiles (Fried and Nesse, 2015). Consequently, in depth investigations of these mental states require opportunities for individuals to discuss and describe their subjective mental states. Therapists from cognitive-behavioural backgrounds are also recognising the importance of reintroducing detailed exploration of subjective experience into both therapy and research (Taschereau-Dumouchel et al., 2022). One of the central ways subjective experience is investigated is with qualitative methodology. Braun and Clarke (2014) point out that “…[*q]ualitative research offers rich and compelling insights into the real worlds, experiences, and perspectives of patients and health care professionals in ways that are completely different to, but also sometimes complimentary to, the knowledge we can obtain through quantitative methods*” (p. 1). The use of interview data was to explore in-depth how participants experienced the intervention. The interviews were conducted via a focus group at three time points after baseline: after the first 12 sessions of CFT, just before the second set of 13 sessions and at the end of the second set of sessions. Due to unforeseen circumstances (an emergency with one of the participants), the second focus group was interrupted, and recording stopped part way through the second question. As the participants provided relevant feedback in a short amount of time, the feedback was included for analysis. Moreover, due to personal commitments, some of the participants could not attend all the focus groups, therefore, six participants attended the first focus group, six attended the second, and five attended the third. As all participants were invited to join at the time of the focus group, the groups represent some of the participants who took part and at a later date may have withdrawn. As the focus groups focused on the impact of the intervention, rather individual outcomes (i.e., case studies), the authors decided to include all participant feedback for analysis as it was deemed relevant. All participants were given pseudonyms to preserve confidentiality.

Focus groups were led by the same interviewer, who used the same interview schedule for all three focus groups. The interviewer was not involved in the therapy, transcription or analysis. The below summarises the questions. Participants readily identified with the questions, although in retrospect it was felt that some of the wording of the interviews may have been initially unclear (and could be improved for future research), for example using words such as ‘psychoeducation’. Moreover, participants shared reflections additional to the specific questions and are included in the analysis.

• ***Question 1.*** This set of questions relate to your experience of the evolutionary model of compassion and how it might relate to your life situation. Can you describe how you experienced this in the psychoeducation?
• ***Question 2.*** This set of questions relates to the exercises and practices. As you know there were a series of exercises and practices relating to breathing, mindfulness and developing a compassionate sense of self and the compassionate image. Can you describe your experience of the exercises and practices, in general? How were the exercises and practices for you?
• ***Question 3.*** This set of questions relates to your general feelings of self-criticism: What, if any, impact might the exercises and materials have had on the way you think and treat yourself particularly at times of difficulty, setbacks or failures?
• ***Question 4.*** This set of questions relates to how this course has helped you in general with issues of mood and emotion. Do you think the course and its modules have in any way changed your approach to how you think about and manage your moods and emotions?
• *The final set of questions* are focused on moving forward and any other feedback. What would support you to grow and further cultivate your inner compassionate self and compassionate mind? Any other thoughts you would like to offer on your experiences, or any insights you would like to feed back to the research team on how to improve the therapy.

### Data Analyses

#### Self-Report Measures

Data was analysed using SPSS versions 26. Results from all six participants who completed the study and completed the self-report measures are reported. Item-level missing data were imputed using the mode for scales with fewer than 20% of items missing. In the case where missing data was higher, last observations carried forward was used to calculate mean scores. Given this was a pilot study with a small number of participants, statistical analyses to determine significant differences were not used and instead, descriptive statistics and visual inspection of scores were considered.

#### Heart Rate Variability

Electrocardiogram (ECG) signals were displayed on a laptop, using AcqKnowledge v. 4.1 (44), digitized at 2000 Hz and inspected offline using Kubios software – Premium version (45). Successive R waves (identified by an automatic beat detection algorithm) were visually inspected, and any irregularities were edited. Data that, after visual inspection, required more than 5% correction were excluded, resulting in missing data at baseline for one of the participants where it is thought that there was poor electrode connection. Thus, the data for that participant was not included in the analysis and only data from five participants was used. As CFT aims to increase vagally-mediated parasympathetic activity, a time domain measure of HRV (Root Mean Square Successive Difference; RMSSD) was then obtained for resting state, and for all the four scenarios at four assessment points (before and after the first and the second set of sessions). The RMSSD reflects the integrity of vagus nerve-mediated autonomic control of the heart (Laborde et al., 2017).

Given the pilot nature of this exploration, visual inspection and a non-overlapping method for analysing the difference between phases in single-case studies was implemented. Such a method is aimed at quantifying differences between two subsequent phases in a single-case design by descriptively summarizing the extent to which data points in the phases do not overlap (Parker and Vannest, 2009). The significance of non-overlap of all pairs (NAP) indexes were calculated for the five different scenarios combining all five cases (Vannest et al., 2016). The R package SCAN was used for all the analyses (RStudio Team, 2015).

#### Focus Group

Data analysis was conducted using the thematic approach (Braun and Clarke, 2006, 2019) as this type of analysis offers flexibility and methodological rigour. This research adopted a deductive approach, where researchers worked with a predetermined framework relating to the research questions. These provided a structure to explore the experience of participants in regard to specific CFT processes. As mentioned in the previous section, six participants attended the first focus group, six attended the second, and five attended the third.

Two of the researchers (JR and HG) with qualitative research experience conducted the analysis. Braun and Clarke’s (2006) six phases of thematic analysis were followed for the three focus groups. JR completed the first phase by familiarising herself with the data and noting initial areas of interest. She then completed the second phase by compiling an extensive list of codes that aimed to capture the rich content of each focus group, before identifying connections between codes across the three focus groups. Codes were designed to match the participant’s own wording in order to maintain a fidelity to the data. These connections, which related to the most significant frequency of codes, became a focus for subsequent analysis. In phase three, JR identified potential themes with the aid of HG. In phase four, JR checked the codes against the theme candidates, and an initial thematic map was drawn up by JR and HG and presented to the research team. These themes were then matched against the research questions to identify potential connections. In phase five, an over-arching story of the data, created in accordance with the research questions but grounded in the data, was identified, and themes were clarified and named. In phase six, the themes were organised in order to present a coherent narrative of the findings in view of the research questions, and relevant extracts were selected.

## Results

This section will explore the key principles of feasibility as suggested by Bowen et al. (2009) in regard to the information elicited from self-report HRV and focus groups measures.

### Demand

Discussions during the initial exploratory session with PG confirmed that there was a great deal of interest in CFT. Participants were enthusiastic and acknowledged that the idea of ‘instability of social rank mechanisms’ as rooted in evolutionary mechanisms that were not their fault was a novel and helpful way to think about their mood difficulties. They were intrigued by the idea that they could ‘maybe’ learn to explore and use compassion motives to be helpful. This was further supported by comments during the focus groups (as outlined below in ‘Going Forward and Suggestions for the Future’ section). Furthermore, as noted in the introduction, these difficulties are relatively common, often with serious consequences to the quality of life of individuals and in need of improved therapies.

### Acceptability

Participants indicated that the therapy group was perceived as helpful and was highly recommended for individuals with a diagnosis of bipolar disorder as well as for more general use by individuals (as outlined below particularly in ‘Going Forward and Suggestions for the Future’ section).

### Implementation

The initial plan was to deliver a standard 12 module program over 12 weeks. However, as participants became very engaged with the therapy, with desires to discuss it with the therapists and each other in detail, it became clear that the time necessary for working with this group was insufficient. Hence, the therapists and participants sought to extend the intervention. The ethics committee was contacted at the end of the initial 12 sessions to request and an extension. This process took over 20 weeks during which time participants did not receive any active therapy but were able to practise some of the CFT exercises they learned during the first 12 sessions. Consequently, the intervention consisted of the original 12 sessions over 12 weeks (14 weeks in total when 2 weeks break due to holidays considered), a 20-week rest period and then another 13 sessions over 13 weeks. Therefore, the 12-module content was covered in 25 sessions over a total duration of 47 weeks when holidays and break (for ethics) considered.

### Practicality

Of the 10 participants who started the CFT group and completed the baseline measures, nine participants completed the first 12 sessions (90%), whilst six participants completed the full 25 sessions (60%), attending an average of 82% of the sessions. One participant withdrew after the first few sessions, another just before the second set of sessions because of work-related reasons, and two withdrew because they were offered alternative support for their needs at the time which may have interfered with the change process. This suggests that the time commitment is acceptable, and most participants were motivated to complete all the sessions.

### Adaptation

The intervention was based on a manual currently being developed for group CFT therapy (Gilbert et al., 2016–2022). In this study, the therapists followed the manual with some adaptations partly due to the fact that these clients, as part of the Bipolar Service, had already participated in a programme which draws heavily on CBT, family-focused therapy and mindfulness-based interventions, called Mood on Track. As such, initially, the mindfulness module was touched on briefly and later expanded to introduce opportunities to practise mindfulness and to facilitate understanding of how mindfulness supports compassion. Moreover, some modules took longer than allowed for, therefore due to time constraints the module on assertiveness, apology and forgiveness was not covered.

### Integration

This study was undertaken in a specialist Bipolar Service and the intention is that it will have an impact on the regular care offered to people with a diagnosis of bipolar disorder. Participants also indicated how they applied knowledge and practices learnt to everyday life.

### Limited-Efficacy Testing

While this was a feasibility study, and not powered enough to detect significant change due to limited sample size, it is worth noting that from visual inspection of individual scores as outlined in Table 2, four of the six participants consistently showed improvements across the majority of self-report measures and maintained them at the final assessment point after all 25 sessions. Improvements in compassion for self and from others, decentring (ability to observe thoughts and feelings as temporary), inadequate self and social comparison were all observed. Two participants showed more mixed results, with improvements on fewer self-reports. Unfortunately, due to COVID-19 pandemic a 1 year follow up was not possible.

**TABLE 2:** Mean scores and indicators of improvements for all self-report measures at baseline, post, follow up, and final data collection point.

Measure	Data collection time								Overall improvement in scores from baseline to final (and any improvement in individual number of participants)
	Baseline (Time 1)		Post (Time 2)		Follow up (Time 3)		Final (Time 4)		
	*M*	*SD*	*M*	*SD*	*M*	*SD*	*M*	*SD*	
Depression (DASS)	6.50	*6.50*	8.17	*6.11*	8.83	*7.39*	8.83	*7.36*	No (3/6)
Anxiety (DASS)	7.67	*6.02*	7.67	*4.32*	8.83	*5.34*	7.17	*5.12*	Yes (4/6)
Stress (DASS)	10.00	*5.10*	9.17	*3.76*	11.33	*5.50*	9.50	*4.09*	Yes (3/6)
Depression (HADS)	6.33	*3.98*	6.50	*4.07*	7.17	*4.79*	7.33	*4.23*	No (3/6)
Anxiety (HADS)	12.00	*4.43*	10.83	*3.66*	11.33	*4.46*	10.50	*4.18*	Yes (4/6)
Decentring	32.00	*4.20*	35.17	*5.67*	35.00	*6.72*	37.00	*5.90*	Yes (5/6)
Negative affect	27.50	*11.74*	23.33	*7.12*	26.00	*10.16*	24.50	*7.53*	Yes (3/6)
Positive affect	51.17	*10.72*	56.00	*14.30*	58.17	*23.22*	53.50	*17.14*	Yes (4/6)
Active (TAPAS)	19.33	*7.58*	18.00	*6.16*	19.17	*7.47*	17.50	*7.50*	No (2/6)
Relaxed (TAPAS)	10.50	*3.73*	12.00	*4.94*	12.83	*4.79*	12.67	*6.25*	Yes (4/6)
Safe (TAPAS)	8.00	*2.90*	8.17	*3.82*	9.33	*3.56*	8.83	*3.19*	Yes (4/6)
Inadequate self	25.33	*6.12*	21.83	*5.81*	21.00	*8.00*	17.50	*6.83*	Yes (6/6)
Reassured self	14.83	*4.26*	18.67	*4.89*	16.67	*5.99*	17.33	*3.98*	Yes (5/6)
Hated self	8.33	*7.84*	6.67	*4.76*	6.67	*5.96*	6.83	*6.71*	Yes (2/6)
Social comparison	52.17	*10.48*	55.00	*13.44*	58.67	*19.29*	59.33	*14.76*	Yes (5/6)
Social safeness	31.33	*9.89*	33.67	*9.14*	34.83	*14.23*	34.50	*12.23*	Yes (4/6)
Self-compassion	58.00	*13.59*	69.33	*14.08*	65.17	*14.91*	74.00	*11.05*	Yes (6/6)
Compassion to others	85.50	*10.01*	83.83	*8.57*	78.83	*13.38*	81.17	*12.77*	No (3/6)
Compassion from others	59.17	11.48	63.50	*8.94*	63.67	*12.88*	63.83	*12.58*	Yes (4/6)

*The italic values are the standard deviations.*

Moreover, results from the HRV measurements and focus group support the self-report findings and the acceptability of the group.

### Heart Rate Variability

**FIGURE 5:**
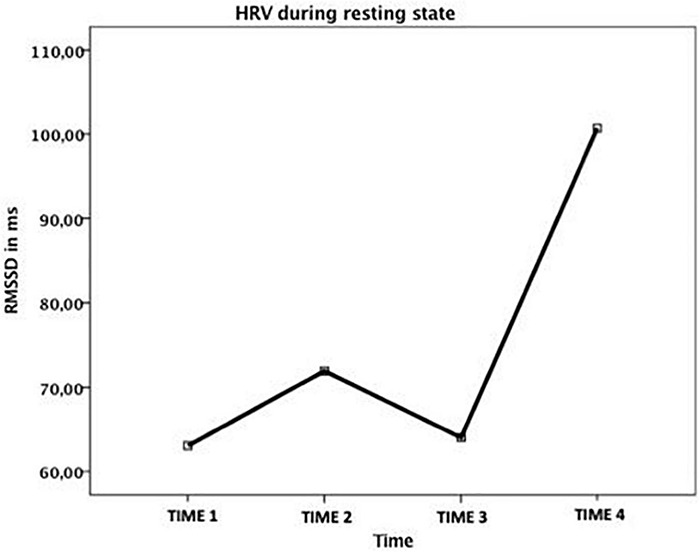
HRV (RMSSD in ms) during resting state at four assessment times.

Visual inspection (see Table 3 and Figure 5) suggested an overall improvement in HRV that was particularly evident during the second set of 12 CFT sessions. In particular, resting state HRV slightly improved during the first set of 12 sessions, seemed to decrease during the “break” between the first set and the second set of sessions, and improved again, more strongly, during the second set of sessions.

**TABLE 3:** HRV (RMSSD in ms) during resting state at four assessment times.

Participant number	Time 1	Time 2	Time 3	Time 4
1	47.30	11.67	39.57	48.99
2	50.93	72.82	61.30	61.30
3	106.42	113.97	98.45	190.89
4	75.77	59.25	83.73	90.00
5	34.97	102.04	37.17	112.35

The overall improvement on HRV was also supported by NAP data analysis, suggesting a significant difference (*p* < 0.05), in terms of non-overlapping of the pairs of the two phases of the intervention for the five single cases series, for resting HRV and two out of four scenarios: defeat, and lonely (see Table 4). Connect and winning scenarios approached significance (*p* = 0.0567).

**TABLE 4:** Non-overlapping data analysis of HRV during baseline and the four different scenarios.

	NAP	*Z*	*p*	CI 85%	CI 90%
Resting state	0.7000	0.0153	0.0153*	0.284 < > 1	0.225 < > 1
Defeat scenario	0.7500	2.5981	0.0094*	0.334 < > 1	0.275 < > 1
Win scenario	0.5500	1.9053	0.0567	0.134 < > 0.966	0.075 < > 1
Lonely scenario	0.6500	2.2517	0.0243*	0.234 < > 1	0.175 < > 1
Connect scenario	0.5500	1.9053	0.0567	0.134 < > 0.966	0.075 < > 1

Non-overlap of all pairs (NAP) analysis of the five different scenarios. We report combined analysis of the pairs of time series (first phase of the intervention vs. second phase of the intervention) per each subject (N = 5). Significance (p) is reported for confidence internal (CI) at both 85% and 90%. * indicates significant NAP.

### Qualitative Analysis of the Focus Groups

The transcripts were analysed in regard to the questions noted in the methods section. For the most part, we have followed the question and answers but occasionally answers to one question were clearly relevant to another question and therefore we connected those themes. Participant dialogue is given in italics. All names given are pseudonyms.

#### Question 1: Understanding and Utilising the Evolutionary Model

Participants found the psychoeducation on the evolutionary model extremely helpful and relatable. The concept that many of the emotional textures we experience are the result of the evolution of the mind and therefore are ‘not our fault’ was found particularly de-shaming and enabled a ‘de-centring and standing back’ and more observational orientation to mental events. A key CFT insight is that we have *minds built for us not by us* and it is that sense in which we understand the concept “it’s not our fault”. This concept highlights the need to learn about the mind in order to take responsibility as noted.

Sophia. *“Because it’s your biology and the way that you’re made, it made us then recognize that some of the things that we went through as a bipolar person, weren’t your fault”.* Another participant, (Jayne) suggested that this knowledge made them ‘…. *feel enlightened, because I felt lighter about things*’ and ‘*more hopeful, more optimistic*’.

Other participants suggested:

Brian. *I think the big takeaway for us was it’s not your fault thing, so these are the things that we’ve done that have just happen to be evolution and so there’s a part of*… *a lot of these things are just the way we’re made. Also, it helped me understand that there might be things going on in my head like anxiety and fear and all these things that actually have a reason or an evolutionary thing, so these are things to protect me and do all that stuff. And it’s helped make me feel distinct from that as in*… *so, that is part of me, but it’s not everything in me and so I can sort of evaluate and sort of talk compassionately to that emotion and sort of deal with it.*

Megan. *And it had quite a profound effect on me when we were doing the meditation and he used the words, it’s not your fault, I had a huge weight lifted and it came out in, in tears, which is how I deal with it. So, I think that consciously it had some affect to letting go of perhaps me feeling like the way I am was my fault.*

Jayne. *So for me, after doing that, it changed my perspective. And, like you were saying, I now relate it to myself, but I don’t blame myself. I think there is a huge anchor when you’re in all the rubbish that you just remind yourself you’re not to blame, it really anchors you.*

Sophia. *Yeah, and that had quite a profound effect on me, just hearing it’s not your fault. Because I think you don’t realise psychologically how much or what life experiences have had an effect on you, but by being part of this group it’s stuff that’s been said that’s actually brought up emotions in me. Or a sense of relief is what I got from the evolutionary side of things.*

Hence, the concept of ‘not my fault but how to take responsible action” encouraged participants not to feel disempowered by their biology but how to engage with their minds and try to take responsibility for their behaviour and actions, how to work with thoughts and emotions and feel ‘more in control’. Examples of this can be seen in statements such as:

Jayne…. *So, it’s easier to deal with situations because, I recognise that there’s my brain function, I recognise that there’s my emotions at play, and the three systems. So, it gives me a choice now, it’s like I can see it quite objectively now, where before, I was just in it and I couldn’t see the wood for the trees, didn’t know how to do this, you know, how to manage uh, my moods, um, thoughts uh, all that self-critic, that inner voice um, but it’s helped me, so I feel more of a whole, I feel more connected to each part and can actually be objective and look at it and think about it.*

Participants noted the value of being able to stand back and separate out and understand their ‘evolved’ emotional dispositions (programming and algorithms). As the therapy progressed, they became more mindful of and less ‘fused’ with the patterns in their minds and to view them more objectively. These insights enabled participants to move forward and de-centre from an over identification with bipolar disorder. Hence, the first sessions gave participants a conceptual framework to think about what was happening in their minds and how to stand back and begin to work with some of the difficult emotions and feelings that arise for them. The evolutionary model helped them consider what ‘their brain might doing’ which helped them to consider thought processes in a more objective, measured way. Examples of this can be seen in statements such as:

Jayne. *I think what I’ve been able to do is uh, uh, from a very fractured, fragmented way my head, the way I work, you know, it’s kind of given it some order.*

Claire. …. *So, I’ve kind of learnt now that you can’t get rid of anxiety, cause we actually need it, because it’s like in our DNA, it’s in our genetics and it’s part of evolution, so it’s more about having to go, okay, I’m going to have to sit with this anxiety and change the relationship with it and how I see it and that’s been a big thing for me.*

Participants also noted how having an evolutionary framework for their emotions which in CFT outlines 1. threat emotions, 2. resource seeking and reward emotions, and 3. secure rest and digest emotions, helped them conceptualise and work with their emotions.

Sophia. *Yeah so that was where, when they described that you’ve got this threat system and a soothing system and a drive, that, we can be going through life and some of those can be perhaps slightly too much of, but what I came to realise was, you’ve got to work on that soothing side*,…. *what we were learning about compassion is if, we build on that if you’ve never known how to sooth yourself, you know, then of course you’re going to be overrun by you know the threat system, possibly and the drive system, so, for me, all that knowledge and it still sticks in my mind today you know? You’ve got those three things going on, and how are they balanced out? You know and I think that kind of gave me that sort of understanding with the condition of bipolar, it’s about a balance you know, in, in trying to balance our moods and it’s the same thing with those three things you know, so that was quite clear to me.*

Megan. *I mean, like I was saying before about emotions, I’m not the most emotional person in the world when it comes to things like this, but I found the evolutionary model for me was like a Haynes manual of the mind. Because before you read those kinds of instruction booklets, you don’t know those things are supposed to be there or how they work. So, now that I know that I am evolved to this place and time right now because I have to be, and that’s okay, I am like, ‘Right, okay, well, anxiety isn’t something to be got rid of’. Like you were saying, it’s something to be there. And now that I’ve read that manual, I know why it’s there and why it serves that purpose, in a way. So yeah, for me, it’s the practical side of the therapy.*

The optimism participants felt as a result of this process helped participants accept that they had a lot of hard work to do but CFT offered ways to build their courage and wisdom to build the confidence to work with difficult emotions.

#### Question 2: Experiences of the Degree of Helpfulness of the Compassionate Mind Training Exercises

The second question sought to explored how CFT exercises helped participants over time to work out what was useful and helpful and when to use these practises. They individualised the practises themselves to make them work with their own brain states.

Claire. *Yeah, I really did struggle with it, and I’m not going to say I still don’t struggle because my attention span is not the best, but again going back to knowing that about myself, that it’s not my fault that my attention span is not very good because I’ve been formed this way, I’ve been like this all of my life. So, if I can only do 3 min of the meditation, that’s 3 min that I’ve got on my side. So, I’m not going to beat myself up because I didn’t get to the end of the meditation.*

Participants initially stated that short exercises were good for them and by focus group 3, one participant explained:

Brian. *I do it in the real world as well now, quite easily. And the way I do it just by not making a big deal out of it, not expecting too much, not expecting magical things, but just getting clear in the mind.*

One participant also highlighted that the exercises had enabled them to access “feeling” states to identify emotional content as opposed to purely engaging cognitively with what was going on for them.

Jayne. *I think that’s the difference with*… *with this course, all elements of it have opened up my awareness of the feeling states. And, for me, when I feel something, I can believe it. When somebody tells me something or I think it, it’s really hard for me to believe. So, if I*… *it’s like, when you have a child, they say children learn through experiential learning, that’s very much so for me.*

Breathing and vagal exercises were seen as aiding emotional regulation by soothing and containing distress. Participants found the idea of ‘helping the body to support the mind‘ by slowing down and grounding themselves helped them cope with difficult situations and events. The awareness of the need to address the links between mind and body were also seen in participants comments about increased use of yoga, exercise and attention to diet.

Of particular interest was the description by one participant who describes the shift in their emotional regulation, after the first 12 sessions, as happening on “another level” but having an influence in their day-to-day life. One of the participants described:

Sophia. *I think when I feel like*… *with these exercises and being part of this whole experience about learning how important it is to give yourself that time and to be kind to yourself and*… *try and be your friend rather than your enemy, your critic, your*… *you know? And it was learning all of that and the exercises, I knew things were happening to me on another level.*

The visualisations offered objectivity that participants found to be a powerful process for them. Some participants found they were able to see and feel what it was to consider different versions of themselves using these the practises, including:

Jayne. *They had a profound affect for me, each exercise, so I have a, I think the compassion to others, I can see their pain, um, so I can see I can help them. When I have any pain and suffering, I can feel it but I can’t see it, so I just want to push it away because I don’t know what to do with it. So, I couldn’t see myself and in one, one of the first uh exercises, I actually saw myself, hug me.*

Later in the same focus group Jayne shared:

Jayne. *Can I just say there’s one visualisation that I use every day*…*and I smile to myself*…. *when he said about smiling at, and we did a visualisation where he said, imagine somebody’s coming towards you smiling at them, and I did it with one person, then it was a group, and then it was me. And it has such a profound affect that it made me feel so good, you know, this other person, I’ve objectified me almost, I can see me, so that was such a wonderful experience, I use that everyday now, I smile at me*….

Visualisation practices were presented in ‘a small step by step way’.

Jayne. *So, I think those*… *because the exercises started off small and simple, it was like building blocks for me, where*… *so I could go there, it wasn’t too overwhelming. So, I think, incrementally, I got to know myself, I got to like myself.*

Shifts and change over the intervention were about embedding these practises to build the psychophysiological structures for the caring social mentality and draw upon compassionate images and safe haven functions to sooth, ground and feel protected. Some participants explained that over time they had internalised them and experienced them spontaneously and effortlessly in their day to day life and at times of need. The shifts and changes that had occurred to embed these techniques were also described.

Sophia. … *being honest, I’d get a lot from the room when we did the actual meditation, I’d go quite deep in the rooms, then I got a lot of reactions and a lot of emotion that came from it, but, I didn’t find it easy to fit it into my life and consciously make that effort, and I know you say it’s great to be able to get it in a little bit here and a little bit there, but I believe what was going on with me was on a different level, it was almost like I was unaware. Because, I’d come up um, in group and realise that actually, I would have given myself a really hard time in that scenario, but I didn’t, so, it was working, maybe I, in a way that I didn’t even realise it was working and it was only when I looked at certain scenarios and situations, that I would normally have that critic there and I’d be feeling oh, you know, that um, I could see that they probably were working, you know and I had got stuff out of it*….

Richard. …*So, I know it’s going to be hard bloody work, but the knowledge I’ve taken in, what I now know, has made it easier for me to accept that hard work and keep going.*

Megan. *It’s a little bit subconscious, I think, sometimes. So, like, you almost don’t think that it made that much of a difference, but then as you were saying before, you kind of almost find yourself doing it.*

Some participants found the visualisation exercises less easy with some reporting frustration with the inability to generate an image (possible aphantasia). Early on some were concerned by disturbing images (tricky brain) and others feeling a pressure to perform the practises in the same way as other members of the group. Some of this was driven by concerns of if they were ‘doing it right’ and what to expect. Nonetheless, participants gradually moved on for this and found ways to personalise the practises. As previously described, one of the participants highlighted:

Claire. *“It’s not my fault my attention span is not very good”* but put this in perspective by saying, *“if I can only do 3 min meditation that’s 3 min that I’ve got on my side”.*

Some participants described being deeply moved by other people’s visualisation even if they could not generate them themselves.

Paul. *I found them hard work. I think*… *especially because a lot of it was to do with, ‘think about this and*…*’, not for everybody but for some people that’s very*… *imagery that they see in their mind’s eye. I don’t have that mind’s eye. So, it was lovely hearing other people*… *beautiful stories. But it’s just really difficult for me to engage with it, I think.*

Brian. … *the angel wings, where there was an image of it enveloping me was not my image, but that stayed with me.*

Participants also reflected on changes that the course helped them make:

Claire. *Like I realised that no matter how much mediation, yoga, you know self-help books, psychiatry, whatever help I had, no matter how much I did, I was always still going to have anxiety, it was just the anxiety might not be as crippling and debilitating and that it might not stop me from doing things, like it’s still there, I get waves of it now, if my new job at work, but you know, I was just going and do my breathing exercises outside for 10 min, then go back in, when I need to I do have medication that I can take from, to get help from anxiety, but I try to do the natural methods more, that we’ve learnt coming on the course.*

Sophia. *I found it very emotional. A lot of the exercises brought up emotion, tears and stuff like that, which I am quite comfortable with now because I know that’s just part of my way to process things. But it’s sometimes still quite surprising when you do the activities. One of the things that came up in one of the exercises was, when he was talking and he just used those words ‘It’s not your fault’, was so huge for me. It was almost like a lift, and the tears, and it all just released. So, on some level, I’ve thought a lot of things who I thought*… *you know, who I am, and just being really. And some of the exercises also got me thinking about the ex-husband, we’d been through a divorce so that was bringing up some more stuff. But the things that I have been fearful of I’m no longer fearful of. Me and my ex-husband, we get on great now, and I do believe that these sorts of things come from within yourself*….

In the final focus group, again Sophia shared:

Sophia. *I think what you’re getting at is that when we came together in the second one we kind of could see how far we’d all come [over talking]*… *oh well, the thought of me talking about my ex-husband was like*… *I was still in so much fear and emotion and everything else to then say, actually, we get on great now. We’re going and doing family events and going to the theatre and able to be in each other’s company, we spent Christmas together. You know, all of those sorts of things, compared to where I was when I first walked through the door, I was like a total wreck when it came to anything to do with my ex.*

#### Question 3: Using Compassion Focused Therapy to Understand and Address Self-Criticism

Participants reported becoming aware of the harsh nature and effects self-criticism during the first 12 sessions and how it had affected their lives. The functional analysis exploration helped them become more aware of the hostility within the process of self-criticism and how to shift into a more compassionate self-reassuring and self-correction and supportive orientation to the self. Participants also found the ‘Stuart video for voices’ (available online under ‘compassion for voices’) which outlines how people can use a ‘compassionate mind’ to work with critical voices very helpful.

Jayne. *The Stuart video was really*… *what that* [Stuart video] *showed me is how I live my life daily, by those voices. So now, I don’t live my life by those voices and I never beat myself up. And yeah, I beat myself up with everything I did daily, everything. Like Sophia, (another participant) it was like I didn’t realise until then how often those voices*…*how much was in there, thick and fast, until I started separating the voices. I’ve got further to develop that. I am much kinder to myself. I never beat myself up, and I’ve got a different relationship now. I am still very depressed and very anxious, but I’ve got a different relationship with it now.*

Sophia. *And the Stuart video for me was quite a profound thing to watch, it kind of just you know, each week, we were getting more and more knowledge, but the Stuart video just simplified it in such a way that I could absorb what goes on and you know that, these voices, or this, that we, that our thought process that goes on, it doesn’t have to be an enemy, you know? And that it’s okay to say alright, well, that voice is fine but, you know, I’m going to carry on today, you know. Its just visualising the way that the video helped me to understand that it’s okay, what does go on in your mind, it’s just how you choose to react to it you know? And that that video you know, for me, I was able to send that to somebody else who’d had lots of different things sent to them in the past, but they found it really profound in the way that, it explains and just reassures you that it’s okay to have these different you know, things coming from the different threat system, sooth system and everything else you know*….

In terms of changing self-criticism and beginning to move towards more self-compassionate ways of relating to self, participants showed the usual fears and resistances. For example losing their ambition.

Brian. *So I mentioned it earlier and stuff, what I think, what was difficult um, was and I’m thinking actually, I’m thinking of um, Richard and I connected on this, was the self-criticism was difficult because, that self-criticism serves a purpose for you. So, it’s like, you get worried about, it’s like if I leave self-criticism, maybe I won’t be doing all this stuff that I know I need to do to survive. If I stop criticising myself, will I then just become ineffectual or something, and so, I think that when you, when you ask a question, it’s like what was difficult? That was a thing that was difficult was reframing that and still feeling sort of secure in it. Does that make sense?*

Overtime participants began to understand that shifting from a hostile self-critical to more self-compassionate orientation could be used to support ambitions and values and to explore and re-evaluate both past and current situations. Important was becoming mindful and noticing when hostile self-criticism had automatically been triggered and then to switch into a compassionate mind orientation.

Richard. *But what I do see is the change in myself, in the fact that I’m far more compassionate to myself than I used to be. Um, when my mind was telling me I was just an arse hole or this or that, I’d go along with that, but now I’m able to tell that to just say no, I’m not, I’m nice. I haven’t done visualisations but rather than smiling at myself, I just try and smile a bit more, you know each day, you know, and, you know I feed myself now, uh, I may have a meal a day and, so on.*

Megan. *So um, it’s always there I think now, I think it’s just saying, well I’ve got two options here, I can go with the behaviour that I used to do, or actually, just be kind to yourself Megan, you know? and, and just keeping it as simple as that, as helped, because the knowledge I’ve been given with how the mind is, is likely to go and work on me.*

Jayne. …. *I’ve got like you know, we had the mantras they told us and I was using them, just a small sentence that you say to yourself, I use visuals and I find I’m using this fishing line a lot to say, right, it’s reeling you in, just let it go.*

One participant highlighted how they were using compassion shifting when working with difficulties with some of the visualisations:

Claire. *Yeah, I really did struggle with it*, [some of the visualisations] *and I’m not going to say I still don’t struggle because my attention span is not the best, but again going back to knowing that about myself, that it’s not my fault that my attention span is not very good because I’ve been formed this way, I’ve been like this all of my life.*

Participants were also able to use their cognitive skills in different ways when they put themselves into a compassionate mind state.

#### Question 4: General Experience of the Therapeutic Process and Impact on Managing Moods and Emotions

While we have tried to keep participants answers to specific questions, there were responses that appeared in earlier questions that clearly related to the overall impact of the therapy. Hence, they are given here. As people went through the course, they changed and deepened their understanding of the evolutionary basis of compassion.

Richard. *So yeah, so it’s helped out, but I, but sorry, had a pretty rough couple, of weeks at the moment though, um, but still, forging on, trying to use it, be more positive, be more compassionate to myself and stuff. I think I mentioned that on the last session or maybe the one before, but the one biggie that I’ve taken out of it is, that self-compassion isn’t selfish. It’s not selfishness, so I’m able now to recognise the people in my life that have just been causing me problems, and making me worse, to feel worse. I can push them back now and just say no. This is my time, this is time for me, I need to fix myself. And uh, and I don’t feel bad about it anymore. I just used to feel horrible and selfish.*

Brian. *But I think it’s, it’s, it’s with that, when you’re like okay, it’s, it’s learning how um, what look, look at the question, what’s the compassionate thing to do? If you preface that with every query, every worry and stuff, it suddenly becomes quite clear, right? It’s like because, as, as because of what (participant) was saying, we established this at the beginning and agree, we’re all quite compassionate people to other people, so actually, we just innately know how to feel about that right? I think it took us a while, some of us to kind of let that guard down and be compassionate to ourselves.*

In regard to the general experience of the therapeutic process, participants expressed that knowledge developed had helped them to identify their ‘brain states’ and be able to stand back and take a more balanced view of things. Overtime participants developed their own ways of thinking about the evolutionary framework for example:

Richard. *The only way I can explain what I took out of this*… *because I am not the most academic person in the world, I tend to just soak things up and then wait for the reaction to come out, and the only thing I could come up with was an analogy that I made up [laughs] to try and explain it to a friend of mine. And that is like, there’s a farmer and a farm hand out in the field and they’ve both got to plough fields. Now, the farm hand has got no idea, he’s just being paid to do a job and that’s the job he’s going to do. The farmer, he’s got to plough a field as well, but he’s got the knowledge, he understands that what the greater gain is going be and the work he’s going to do. But it doesn’t change the fact that he’s got to do hard work to get there. And this is the only way I can understand it. So, I know it’s going to be hard bloody work, but the knowledge I’ve taken in, what I now know, has made it easier for me to accept that hard work and keep going.*

Participants highlighted the importance of helping them to change the way they think.

Sophia. … *you can’t really help what other people do, what you can help is what you think about, what they do. And the same with um, you can’t help what happens to your life, but you can change the way that you think about what happens to you.*

Participants slowly developed their confidence to regulate their emotions using compassionate wisdom and courage to work out how to generate an alternative (non-impulsive) way of thinking and acting and making choices differently. By the third focus group some participants were addressing behavioural dispositions with compassionate self-reassurance and support. For example,

Megan. *So, the compassion that I give to myself now is that accountability. If you go and spend that 50 in wherever, what have you got to do afterwards?*…. *Is this the compassionate thing to do? It’s the indulgent thing to do, without a doubt, but is this a compassionate thing? So, it’s that questionable thing I have all the time, it’s almost given me my conscience back a little bit. So much so that I’ve lost two-and-a-half kilos [laughs] just purely by going, ‘Who do you want to be? Your compassionate image’. I look at that and I go, ‘That’s who I want to be now’.*

Richard. *I’ve certainly out of my shell, and I’ve had maybe four or five people I know point out that they’re actually starting to see my personality come out again. After 3 years of being in the dark, I’m starting to come back to life a little more*……. *And although I couldn’t see it working, it started working. And I can see the result of it now. Well, learning about the way the brain operates is fantastic, because I didn’t have that before, so having that is quite important. Even though I say I’m not an academic person, I tend to just let it come in and see what comes back out. And what’s come back out has been much more positive than when I first started, so I’m quite happy about that.*

Participants described how they started to put their thought processes into perspective, build confidence in the ability to change unhelpful habits, and embed CFT into their lives. Participants began to realise that they could work towards a “new” version of themselves.

Sophia. *It is, because it’s a mental pattern, and this is, I think, again from the evolution thing. It’s the way our brains are formed and the way that we think becomes our norm. So then, all of a sudden, you’re in a group situation where they’re explaining stuff to you about how the brain works and that, but you can change it. Now, that’s huge as well because you don’t think you can, you just think what I am and who I am and how I think is just what it is. But, being presented with information that tells us, actually, by doing certain things you can change that habit. And it takes time, because you’re so used to that default possibly being negative, or beating yourself up, or being a critic, not being nice to yourself. And then, this information is kind of presented in a way like, ‘Oh my God, there’s another way to look at this’.*

By focus group 3, some participants had embedded CFT exercises into their lives and developed skills and competencies to manage themselves. In this extract a participant describes how supported they feel by their compassionate image,

Claire. *But I find the compassionate image now, for me, is the one that’s with me all the time. I don’t even have to tap into it anymore, it’s this little lady who is cheering me on all the time. And she’s there, and I know she’s there. I don’t even have to take my breathing down it feels like she’s on my shoulder sort of thing.*

Hence participants had moved from using CFT to try to help them with their emotional difficulties to it becoming an integrated identity and way of living. This is quite fundamental to the CFT journey.

Of particular interest in regard to the changes over the three focus groups, was the acknowledgement of and changes in the fears and resistances that participants had been dealing with over the therapeutic process. Fears included those of losing creativity and being frightened of ‘being attentionally present’. This acknowledgment was more noticeable in focus group three. Participants became more comfortable discussing resistances once they felt that they had ways to work with them, their competencies had developed, and their emotion tolerance had become stronger.

Brian. *There’s another fear*… *another fear I had was if you looked at these things and broke them down, I might lose a part of me, my creativity might go and all of that might be tied up in that unanswered question. I don’t think that’s true now. I think that actually shining a light on everything and doing this can*… *does not kill off the creativity, does not kill off the magic. And I think I’ve always wanted that, but now I’m like, ‘Oh, that is totally possible’. Because it just is. So, it’s literally this whole idea of being present and being like, ‘What’s going on right now?’ And it’s not like*… *before, I think I was kind of sometimes really scared to do that, especially when I was going through something traumatic*…… *But now, I feel confident that I could deal. you’re still going to be in that situation, it’s kind of going back to what we were doing there, but the idea of being awake in the now, even if it’s not a perfect situation, is the perfect part. So, I am still me, I’ve got all these tools, I’ve got this compassionate way of talking about things, I can deal with everything, so it’s fine now. So, it’s really been that kind of being able to be present and have access to everything, and it’s not some kind of magic bipolar thing going on making me a genius or any sort of stuff.*

Participants were very moved by the developing group dynamics over the course of the intervention. Whilst this is the intention of group therapies, CFT specifically focuses on the importance of sharing and caring for each other in the group as part of developing the care focused social mentality. Participants expressed how powerful it was to hear others talk about issues that they themselves had internalised, and this included elements of shame that had felt to be self-isolating. As one participant expressed:

Brian. *Everybody in this room have said things that I would, made me feel less lonely right?*

Group members acknowledged how they had been able to develop mutually supportive, prosocial and caring relationships with one another and the therapeutic process encouraged them to address difficulties with communication with others in their lives (outside the group) and become less fearful of doing so. Over the three focus groups participants seemed less competitive with a shift towards the building of trusting and affiliative relationships. One participant stated,

Brian. *I do find that I have an idea and I think, ‘Oh, I wish I could tell that to my people’*……. *the second that I recognised it means I know it was good enough to share, and so I will just execute it and stuff.*

Participants became encouraging and supportive of one another over the course of the intervention, creating a secure base and safe haven. They came to trust the judgements of each other.

Sophia. *You know, the period that I’ve just gone through, I could quite easily just say, ‘Yeah, it’s all to do with my bipolar and it’s this, that and that other’. But actually, no, (self), she then comes back at me with all this stuff that I know in my head, but I need it validating from somebody else. And I think that’s what has helped in this group as well, because, however, we’ve been thinking or whatever our mood was that particular day, we’ve come in and by hearing what you’ve got to say, it makes me feel a bit more*… *because we can all relate on the bipolar side of things, but we can also go along this journey learning about how to be compassionate. But then also, like you’ve just described, be able to differentiate between the two is not always easy. But hearing others talk, you then figure things out, don’t you, in what people say?*

Some participants noted changes in how they related to their bipolar condition.

Claire. … *I don’t know about everybody else, but whenever my mood changed or something happened it was, ‘Oh God, why me? Why me? What have I done to deserve this now?’ There’s none of that anymore because, actually, it’s just life. We were just saying before, life is there and it will hit you in the face every single day of the week. The course has made me realise that now, that a lot of that isn’t bipolar. Bipolar is the way that I look at things not the way things are. So, it’s very much separating the two for me, which has then made instantly my mood more settled and manageable.*

Another participant (Megan) shared in the first focus group how participating in the group changed how she related to her diagnosis and *‘accept her condition’*

Megan. *I just wanted to say as well, going through this experience for me, with people who have got bipolar, it has really helped me because I was recently diagnosed with it, so hearing other people talking with the same condition that I’ve got, I got a lot of you know, feeling of identification with others which was really quite helping me to accept my condition, as well as what I was learning on this course as well, you know. Identifying with other people was huge for me, identifying the way they thought about what they’d experienced on that exercise you know, there’s somebody in this room that feels the same as me and acts the same as me and the same struggles as me, and that we’re all learning together to be better and that was, that was huge as well.*

This continued and by the third focus group, Megan shared how she was able view her diagnosis differently.

Megan. … *I think I’ve been able to separate myself more from it. Originally, when I met people, after having my diagnosis, I used to almost feel like I had to kind of tell them, ‘Oh, I’m bipolar, I’ve got a*…*’. Now, I kind of separate myself, it’s like I am someone who suffers with bipolar disorder, but I am well at the moment and haven’t had any episodes for 18 months’, or whatever. So, I think it’s important to separate the two out and not to blame it for everything.*

Participants were concerned that people felt supported when the intervention had finished, and the group were no longer meeting up. Some group members set up a WhatsApp group so that they could stay in touch and one participant described how he would use this platform to remind participants of a powerful image created by one participant which had had a shared emotional impact:

Brian. *And all I can say to you, I was like, ‘I’m not going anywhere, you’ve got me. If you want me, I am here forever’*……. *I’ll remind you*… *I’ll remind you about the wings.*

The reference to the wings was that one of the participants had imagined their compassion image having angel wings.

Participants also reflected on changes that the course helped them make:

Claire. …. *I haven’t taken diazepam the whole time I’ve been on this course*… *So, that’s a massive thing, as well as having a low mood, yes I have gone back on my anti-depressants, but the anxiety, you know, the panic attacks, I’ve been stopping myself from getting to that panic attack phase by going, you don’t need this right now [name of participant] just get out, it’s fine, just leave, walk away’.*

Megan. *I think the therapy*,…*has stopped me from catastrophising everything. Whereas before, he said, ‘You’ve got depression’ and that was it. It was a write-off; the day was a right-off. So, this therapy has stopped me from catastrophising everything, making everything bad instead of just that one thing, that we can deal with in a chunk, that kind of thing. I just wanted to add that onto the last bit [laughs].*

#### Question 5: Going Forward and Suggestions for the Future

Over the course of the focus groups, participants reflected on changes that the CFT group helped them make and the impact it had had on them.

Jayne. *I was in such as bad state when I came here, because I nearly didn’t yeah, such a bad state, suicidal, um, so to go through the course, I was absolutely 110% committed, yeah, to get here for me. Um, so its quite selfish, I thought, I’m going to take from what, this is kind of [overtalking] almost like my last chance, I’m 54, I’m so tired of procrastinating, so tired of you know just not being able to progress, um, that to then go through it and have some self-belief some self-worth, I could sooth myself, um, to then starting a business, I will never ever let go of that self-compassion, because I, I think I’ve been very lucky, in seeing how if I use it, uh, what a profound effect it can have in such as small time.*

Sophia. So*, it could be something nicely goes from (previous intervention group) to*… *[over talking] then you’d have the same sort of people that have gone on journeys with (previous intervention group) to go to the Compassion Therapy and*… *just all that information and knowledge about the condition. And then there is this positive way of looking at yourself. Because I think people with bipolar are often particularly hard on themselves, because you don’t understand what the hell is going on with you. You know, one day you’re like this and the next day you’re like that, and nobody can tell you why. And you always think that there is this*… *there’s got to be this outside reason that’s caused it. But until you actually start to get well, and you go on these programmes of self-discovery of yourself and how you actually work, and you’re learning to not feel bad about yourself, it’s huge. It’s completely turned my life around.*

Sarah. *I didn’t expect this to be as effective as it was. I was thinking it was just something to make an influence sort of thing*… *well, it’s extremely useful, you know?*

There was general agreement that participants wanted to stay in contact with each other and the service. Participants described how positive their experiences had been in relation to group cohesion and how continued contact with fellow participants could provide reminders, grounding and continuing support for one another. It would also provide an opportunity to “check in” with therapists to see how people were getting on.

Megan. *Um, I think what would be really helpful is if we could have meet ups*… *Where we can meet up and, and somebody there to sort of say, well my, how, how are we getting on? us know? It’s, it’s almost like a reminder.*

Richard. *But having like a, a continuation of some kind, meeting up with people, it’s like a grounding, it brings me straight back.*

Jayne. *So yeah, for me, um, that’s it now, I will always be self-compassionate, but I think it would be nice to have, like a refresher meet up course, um, just to see how people have developed or where they have got stuck and will have actually learn new things about our own self, self-compassion that we can share*….

Claire. *I think we all said before, didn’t we, that maybe a bi-annual*… *like, a kind of six-monthly meet up or whatever*… *[over talking] yeah, that’s*… *I mean, we could all go for a coffee and stuff, but obviously if there was something to facilitate it every 6 months, or maybe once a year or whatever*….

Participants were very interested to learn about the results and kept informed about the outcomes:

Megan. *I’d like to see exactly what benefit does come out of it*… *And actually, I think just to be kept in the loop would be nice. For instance, this research is not going to end, is it? It’s going to go on. And if they do start trialling it again with different people with bipolar, maybe a yearly newsletter about what’s been going on. I know that the*… *I’m part of a research programme with a university, and they send me a newsletter about what they’re doing. And I find that really insightful.*

Brian. *I want to see my heart rate data.*

Sarah. *We’re all little guinea pigs and we’d like to be kept in the loop [laughs].*

By the final focus group, participants had started to think about how they would individually continue integrating CFT into their lives*:*

Sarah. *We’ve all taken our bits from it, we’re all*… *we’ve all got to make this work for us, and we’re holding onto that bit. We might not have held onto everything, but we’ve got something from it.*

Megan. *I think it’s like a language, isn’t it? You’ve got to use it or actually lose it? So, we have to condition ourselves to be reaching for the compassion first before anything else, every time there’s a crisis or an emotion that we feel uncomfortable with, perhaps. And I think it’s training yourself to do that.*

Suggestions were also made as to what materials would be useful for continuing their practices and the need to make resources easily available and in different formats.

Sophia. …*I was just saying to him it would be great to have a bit of a summary almost, for some of the things we’ve said each week.*

Megan. *…we’ve had a lot of stuff come through various emails, but I find it hard to know where to go just to find … looking for one and I can’t find it, so I don’t know if there is some central place where they all are.*

This a helpful observation that clients could be given workbooks that collate the materials, such as psychoeducation and practices that were given week on week, so they are able to refer back to them in a single document and as a record of their process. This could also be stored on a webpage for clients to access with links to other useful information for them.

Participants felt that CFT should be offered to various groups and suggested how it should be offered.

Claire. *I think it should be offered to people with bipolar.*

Brian. ‘*Okay, if you can go with*… *will it apply to everybody?’ But I think, honestly, it will.*

Megan. … *instead of them going, ‘Right, here is a prescription for fluoxetine or sertraline or whatever they give them, going, ‘Have you heard about Compassionate Focused Therapy?’ Because I think it would do you the world of good. I don’t believe that you even necessarily need to have a massive experience of mental health services to benefit from this. I mean, I’d like to see compassion focused therapy in church halls everywhere. Because people that are under stress, people are under family pressures, bereavement, it works, it’s an all size fits all therapy.*

### Therapist Observations

Over the course of the intervention, the therapists made notes of their observations. In general, they map onto feedback from the focus groups. They observed that the group found the compassionate mind training practices emotionally powerful and helpful. Gradually over the weeks, they observed the way participants came to internalise their compassionate images, experiencing them spontaneously and effortlessly. The ‘compassionate self’ practice also appeared to inspire compassionate behaviour outside of the group. For example, one person found the courage to be assertive with their mother, something they had always found difficult. For others, there was a sense of growing perspective and developing a wise authority over other parts of themselves, captured by one person’s metaphor “I’m becoming the farmer rather than the farmhand”.

Playful exploration of how rank-focused and competitive attention, thoughts and behaviour show up in everyday situations enabled the participants to show greater acceptance of, and insight into, their competitive mentality. Noticing social comparisons and thoughts relating to inferiority and superiority became a specific form of mindfulness practice. The therapists began to invite participants to deliberately stimulate the ‘competitive self’ and then to switch to the ‘compassionate self’ and to observe the effects. Once again, participants reported “profound” insights and changes in their social behaviour, such as shifting from experiencing sibling rivalry to a sense of gratitude for the relationship and relating less defensively to others and noticing a greater sense of connection emerging. One participant described how experiencing frequent social comparisons often set them up for unrealistic goals which lead them to feel pressured, angry and anxious. Switching to their ‘compassionate self’ created quite profound changes in this pattern of activity; helping them move away from being focused on keeping up with others to a focus on what was in their best interests in the moment. They gave an example of how one day they felt pressure to cook a fish pie but in a crowded supermarket they couldn’t find the ingredients, felt frustrated, anxious, guilty and angry. They said that usually this would lead to a “hulk-like outburst”, however, this time they found that they had allowed themselves to let this go, make another meal instead and their family had a pleasant mealtime instead of one full of anger. A few days later, they cooked a fish pie. This may reflect a natural shifting from a competitive mentality focused on keeping up with others to a caring mentality, where they could focus on what was in their best interests in the moment.

The group watched clips from the comedy sketch show ‘The Fast Show’, which regularly depicted a character called ‘competitive dad’ who is stuck in his competitive mentality even in his family life, with obvious humorous results. This enabled the group to identify different thoughts, feelings and behaviours that were typical (if exaggerated in this example) of the competitive mentality. One person, who did not previously identify themselves as competitive, began to reflect on how they were often triggered into this mentality by one of the other mothers during the school run and that this relationship was a source of stress to them and one they wished to avoid. Following the motivation switching exercise, they began to explore the possibility of trying to stay with their compassionate self when next on the school run and they set this up as an experiment and the group were excited to see how it went. The next week they reported that they were able to stay aware of this dynamic and hold their compassionate self and that to their surprise their interaction began to take on a new feel, with both seemingly acting calmer and more open to the point where they began to enjoy it. Over the weeks they found that this relationship had fundamentally changed for the better.

The ‘*Multiple Selves’* exercise, where the emotions of the threat system (anxiety, anger and sadness) are isolated and explored as separate ‘selves and brain patterns’, was a major revelation for most participants. They generally had not experienced therapeutic work focused on differentiating and deepening insight into their emotions and initially felt anxious about engaging with them. One person noted that they always tended to feel very out of control “when emotions turn up” and others struggled to differentiate between anger and anxiety and between sadness and depression. After the exercise, one person said that they “had never considered emotions in this way before” and, in regard to anger, previously thought that “I just shouldn’t feel it”. By session 13, one person noted that they were now better at tolerating their ‘anxious self’ and at not letting it dominate their behaviour. They were much more able to take positive opportunities such as applying for jobs and attending social events that they would otherwise have avoided. Multiple Selves also helped participants engage usefully with depressed and manic states, rather than just monitoring their presence or absence. This was particularly significant because, as one group member stated, prior to this exercise “compassion had never been present when either depressed or high”. Overall, the feedback was that the multiple selves exercise helped them to differentiate and understand their emotions better, link to emotional states that they had feared and to tolerate them. This in turn led to important changes in their overall emotional balance and behaviour.

The group were generally very engaged with the material and very enthusiastic to share their own experiences, which led to many rich and meaningful exchanges that really drove the group forward. However, after a few sessions it became apparent that both facilitators and the group members were increasingly vying for limited space to talk. This competitive dynamic showed up as interruptions when others were speaking. At this point it was important for the therapists to playfully acknowledge the dynamic, de-shame it as a natural process and to try to re-set the boundaries. It was helpful to then cue the group into mindful attention of this process and then to grounding in the body and switching to a compassionate intention. It was necessary to repeat these prompts, and this remained a challenge throughout the sessions, albeit to a lesser extent in the second part. On reflection, the initial large group size and limited number of sessions were likely to have contributed to stimulating this competitive dynamic. The therapists found that the initial arrangement of 12 group sessions was inadequate both in order to cover the material and to deal with the inevitable fears, blocks and resistances that are key to the therapeutic process in CFT. It is highly recommended that future groups are of at least 24 sessions and ideally 30–40 sessions, as this is likely to enable a slower pace and more repetition of key practices and concepts and enable some of the psychophysiology changes (e.g, to the vagus nerve) to become more embedded.

## Discussion

There is good evidence that psychosocial treatments have an important role to play in the support and management of people with a diagnosis of bipolar disorder (Vieta et al., 2018; Davenport et al., 2019; Miklowitz et al., 2021). In addition, as a transdiagnostic therapy, and as noted in the introduction, CFT has a range of benefits for people with complex mental difficulties (Gilbert and Simos, 2022). For example, Brown et al. (2020) and Forkert et al. (2022) found that compassionate imagery has significant impacts on people with paranoia beliefs and persecutory delusions. This paper outlined the basic science of the compassion and evolutionary approach to bipolar disorder and explored the experience and effectiveness of CFT for a small group of clients with a diagnosis of bipolar disorder. As a pilot study, using the criteria as suggested by Bowen et al. (2009), the results provide promising evidence of its feasibility and acceptability. Although this was originally designed as a 12-session intervention, it became apparent that for this group, this was too short to enable the complex processes of the therapy to unfold and become embodied. Hence, with the seeking of ethical approval, which took 20 weeks, the therapy was extended by another 13 weeks, giving a total of 25 sessions over 47 weeks (which included a 2-week holiday during the first set of sessions). This generated some unanticipated, and important insights. This 20-week break gave participants opportunities to further develop their compassion practises and insights in the knowledge that they were coming back together again. In the final focus group, one of the participants reflected on the break *“I think what was really important was having these two parts of the course and having the break in between was very impactful because it was like they took the training wheels off, so you had this period where we weren’t seeing each other each week, and then we came back a couple of months later”* (Brian). Indeed, the importance of continuation of support is a repeating theme in these kinds of groups. We suggest then that therapeutic holidays and breaks that can significantly extend the time for therapeutic engagement may be preferable to uninterrupted therapeutic journeys. Our experience with groups with complex difficulties suggests that because of the biopsychosocial changes CFT is seeking to generate, these extended periods of time provide opportunities for embedding physiological and psychological change, thus rendering them more effective.

### Self-Report

Our data using self-report measures are equivocal. Similar results have been found for other studies suggesting that self-report measures of change in people with a diagnosis of bipolar disorder can be sensitive to high levels of short-term variation (Jones et al., 2018, 2019). Hence, given this was a feasibility study with a small number of participants, indicators of change are reported for each variable. The results offer interesting indications that generally participants improved in their self-report. Four of the six participants consistently showed improvements across the majority of self-report measures and in particular improvements in compassion for self and from others, decentring (ability to observe thoughts and feelings as temporary), inadequate self and social comparison. Interestingly, although there was an increase in some individual compassion for others participant scores, overall, there was a (small) mean decrease from baseline to the final data collection. This may have been partly due to the changes in the way some participants viewed social relationships as noted by the therapists. One of the participants, for example noted ‘*I’m able now to recognise the people in my life that have just been causing me problems, and making me worse, to feel worse. I can push them back now and just say no’* (Richard). These statements indicate that participants had recognised that compassion involves appropriate assertiveness. Two participants showed more mixed results, with improvements on fewer self-reports which may have been linked to life events at the time of completion. Nonetheless, we note these two participants showed some of the most striking improvements in baseline HRV, as they both shifted from being in an ‘at-risk’ profile of HRV (of cardiovascular disease), to a healthy profile of HRV.

### Heart Rate Variability

Analysis of heart rate variability suggested that the CFT intervention can be effective in improving participants’ vagal tone (parasympathetic activity) both during resting state and in response to the connectedness scenario. In particular, visual inspection suggested an overall increase in resting state HRV, indicating that CFT, originally designed to stimulate caring motivations, seems to produce a more balanced interplay between the sympathetic and parasympathetic divisions of the autonomic nervous system, and more adaptive, flexible patterns of psychophysiological functioning. This is important, given that in comparison with healthy controls, patients with a diagnosis of bipolar affective disorder tend to show lower resting HRV (Chang et al., 2014; Faurholt-Jepsen et al., 2017 for review; Henry et al., 2010; Quintana et al., 2016), indicative of an increased risk of cardiovascular disease (Goldstein et al., 2015).

The literature on the differences in baseline levels of HRV between healthy controls and patients with depression seems to suggest that a 5 ms change in RMSSD could be used as a measure of reliable change following an intervention (Moon et al., 2013; Hu et al., 2016). Indeed, RMSSD provides an index in a common metric (milliseconds) and is well suited for use as a biomarker in a clinical setting (Jarczok et al., 2019). For all participants in the current study, the majority of their improvements in resting HRV far exceed this value, suggesting that CFT results in a clinically significant improvement in HRV.

Visual inspection also suggested that, overall, participants’ HRV improved in response to both positive and negative scenarios relating to competitive and affiliative motivations. This seems to suggest that participants improved in their ability to engage with (rather than avoid) both pleasant and unpleasant emotions (Steffen et al., 2021). Moreover, results of non-overlapping analyses suggested that CFT was particularly effective in improving participants’ HRV in response to negative scenarios (defeat and loneliness). This indicates that participants, in particular at the end of second set of CFT sessions, were more able to regulate their psychophysiological responses to threatening scenarios of defeat and loneliness. Moreover, authors noted that when inspecting individual scores four of five participants showed improved HRV reactivity specifically in relation to the connection scenario and this approached significance for the non-overlapping analyses.

### Focus Groups

We conducted three focus groups: at the end of the first 12-week block, and then at the beginning and end of the second set of sessions which came after a 20 week break whilst seeking ethical approval for extension. We were interested in five questions pertaining to the experience of the therapy as given in the methods and results section. The focus groups generated a range of different core themes to their experiences of CFT.

#### Understanding and Utilising the Evolutionary Model

In the pre-therapy invitation presentation by PG and also in the first therapy sessions participants were introduced to the evolved nature of the mind and the possible evolutionary (social rank-affect regulating system) underpinning of bipolar disorder. Participants indicated that the evolutionary model helped them consider the nature of bipolar disorder in new ways and also to recognise that the emotional difficulties arising from it were ‘not their fault’. Some participants referred to the impact of this as ‘huge’. This enabled them to consider that their susceptibility to mood switching maybe related to an evolved brain state regulation system that we all have, but which for them has become intensified and dysregulated rather than ‘just an illness’. For example, winning multi-millions on a lottery can put people into hypomanic states and equally major losses in one’s life can trigger depressions. Furthermore, the process of intensification and dysregulation may relate to trauma as well as genes but in both cases is ‘not their fault’. This insight also helped them to ‘stand back and de-centre’ from their bipolar condition. For example, one participant noted *“because it’s your biology and the way that you’re made, it made us then recognize that some of the things that we went through as a bipolar person, weren’t our fault” (Sophia).* Holding the evolutionary idea that our brains have been ‘built for us not by us’ and the brain is not well-designed as such (Gilbert, 1998; Nesse, 2019) supported the participants in their work with self-criticism, shame and mindfulness (see below). Although therapists did not go into a lot of detail about neuroplasticity, and the nature of the autonomic nervous system, it was sufficient for participants to see their issues through the evolution informed biopsychosocial approach including how different motivational systems function and how practices can help people rebalance their motivation systems to go with compassion motives and processes.

Introducing participants to the three affect regulation systems, with a specific focus of understanding the functions of different emotions enabled them to think about their emotions in new ways and then to recognise and accept their emotions and work with them using a compassion focus. Over the three time periods participants noted they were becoming more competent in differentiating, tolerating and working with their emotions.

#### Experiences of the Degree of Helpfulness of the Compassionate Mind Training Exercises

This question generated many themes, particularly around the value of practicing soothing rhythm breathing, and the different dimensions of the visualisation. Participants liked the embodied practices such as the deliberate use of body posture, voice tone, facial expressions and the concept of ‘helping the body to support the mind’. The breathing exercises were experienced as aiding emotional regulation by soothing, slowing down and grounding in the body to help with distress and to support coping. Equally participants recognised that compassion was not just about soothing but also required courageous action and addresses needs (Di Bello et al., 2021). The awareness of the need to address the links between mind and body were also seen in participants’ comments about increased use of yoga, exercise and attention to diet.

In regard to visualisation, practises ranged over different dimensions of safe place, compassionate other and compassionate self. Some individuals found them difficult (possibly with degrees of aphantasia; see Zeman et al., 2015) and at first some experienced intrusive unwanted images. When generating a compassion image one group member visualised an ‘Angel with wings’ and this became an image that others in the group adopted and found helpful. The use of *shared compassion images* was noted in earlier studies too (Gilbert and Procter, 2006). Such images can be seen as tapping into internal processes for creating ‘a secure base and safe haven’ (Gilbert and Simos, 2022).

A number of participants recognised that they often monitored and worried if they were doing the exercises ‘right’ and if other people could do them better than they could. This was playfully noted as partly an example of ‘social rank thinking’ and to use their own experience of helpfulness. However, over time they found their images and visualisations increasingly automatic and useful. A common theme was that it was best to do the exercises ‘little and often’.

In CFT mindfulness is used to become an observer of the contents of one’s (evolved) mind in order to work with its brain states and content rather than engaging in longer practises. Even though participants had prior knowledge and training in mindfulness, some still found it difficult. However, over time this generally improved for participants. One participant described their experiences of mindfulness in the following ways: “*I get very uncomfortable”, “my brain is throwing stuff at me” -* (Richard). Another participant described their resistance to mindfulness and how they had to work hard to find the courage to overcome it and *“engage with the present moment”*. Some of the initial thoughts they had to work to overcome included, “*no I don’t want to do that, “this is going to kill me” and “I’m scared of the last bit*” (Brian). However, as their courage developed, the participant stated that mindfulness had “impacted everything else” (Brian) and that they were seeking online support to develop this practice. Some found the compassionate mind practises difficult, confusing and at times resisted them, but with practise and gentle support from the therapists, participants were gradually able to ease into them and to experience their benefits. Shifts and change that occurred over time appeared to become more embodied and automatic such that participants were able to draw upon compassionate and safe haven functions to soothe, ground and feel supported. Some participants described that by the end of the intervention they had internalised these visualisations and experienced them spontaneously and effortlessly in their day-to-day lives and at times of need. These shifts and changes were also described as “a little bit subconscious”, “you almost don’t think that it made much of a difference”, “you just find yourself doing it” (Megan).

#### Using Compassion Focused Therapy to Understand and Address Self-Criticism

Compassion focused therapy suggests that hostile forms of self-criticism can operate as one of the most important recursive loops into the threat system. A number of participants indicated that the therapy helped them recognise the hostility of their criticism (which they hadn’t before) and the value of switching to a more compassionate focus for self-support, self-reassurance and how to accept setbacks. Key here was helping clients understand that compassionate self-reassurance and self-correction are not simply easy options or ‘cop-outs’. Some feared that without hostile self-criticism, they would lose their identity, drive, creativity and ‘security’. As one participant described: “*self-criticism serves a purpose for you. So, it’s like, you get worried about, it’s like if I leave self-criticism, maybe I won’t be doing all this stuff that I know I need to do to survive. If I stop criticising myself, will I then just become ineffectual or something, and so, I think that when you, when you ask a question, it’s like what was difficult? That was a thing that was difficult was reframing that and still feeling sort of secure in it”* (Brian). Fears of toning down or moving away from harsh self-criticism are common in many mental health problems (Gilbert, 2009; Pauley and McPherson, 2010; Tierney and Fox, 2010; Lawrence and Lee, 2014). For some clients who have a long history of harsh self-criticism, the idea of cultivating compassion towards themselves feels alien and untrustworthy. It takes time to address these and other fears of compassion that are strongly linked to mental health problems (Kirby et al., 2019). These issues were also found in a qualitative study, by Steindl et al. (2022). They asked 64 therapists how they perceived and worked with FBRs. The four key themes that emerged were 1) getting ‘behind’ the FBR by understanding its function and linking it to the past, 2) working with the psychoeducation of ‘tricky brain’, normalising FBRs and ensuring clients have a clear understanding of the CFT definition of compassion as an algorithm, 3) using experiential interventions such as soothing and safeness in the body, compassionate imagery and chair work, 4) respecting the wisdom of FBRs as safety behaviours and slowing down and recognising the complexity of FBRS, and collaborating.

Recognising that compassion, self-reassurance and self-correction built internal courage to face difficulties supportively was an important step. Both could actually improve one’s drive and creativity by building confidence and improving coping when faced with setbacks. One participant suggested that “…*it takes time, because you’re so used to that default possibly being negative, or beating yourself up, or being a critic, not being nice to yourself. And then, this information is kind of presented in a way like, ‘Oh my God, there’s another way to look at this”* (Sophia). Many participants recognised that the evolutionary model significantly helped them to step back from self-criticism and soften their self-attacking. Some participants became mindful of when they were self-attacking and then switched to their compassionate image or own mind state. One participant stated: “*so, the compassion that I give to myself now is that accountability. So, it’s that questionable thing I have all the time, it’s almost given me my conscience back a little bit. Just purely by going, ‘Who do you want to be? Your compassionate image’. I look at that and I go, ‘That’s who I want to be now”* (Megan). These examples offer insight into the way the different components of CFT work together, as in this case identifying hostile self-criticism and then recognising one wants to have a more compassionate orientation to oneself and others.

#### General Experience of the Therapeutic Process

As for most group therapies, participants acknowledged that the group process itself was an important process supporting change, particularly for addressing self-criticism and shame (Carter et al., 2021; Cattani et al., 2021). CFT focuses on the three flows of compassion and it was heartening to see that in addition to working on self-compassion, a major component for participants was the sharing of compassion with each other and the feeling of not being alone, and that compassion was something that could be shared. They acknowledged beginning to experience a different type of social connectedness and at the end set up their own WhatsApp group to support and help each other using compassion approaches if others were struggling or lonely.

Participants returned again to the fact that the evolutionary model of understanding (that so much of our mind is built from evolved algorithms that we don’t and can’t choose) was a major new way of thinking and a relief; not to see bipolar just as a disorder, but as linked to an evolved (motivation) process and be able to stand back from it and engage with the compassion motivation to it. This was a very de-shaming experience. Important too was understanding what compassion was and wasn’t in terms of building courage and wisdom and not a ‘cop-out’. In addition, there was some evidence that participants shifted from trying to be helpful to others to be liked, which is called submissive compassion (Catarino et al., 2014) to more genuine compassion.

Although trauma was not specifically addressed in the groups, a number of participants indicated how engaging with self-criticism commonly stimulated issues of trauma but, taking a compassionate orientation helped them with their experiences of trauma. Participants reported becoming more aware of the potentially negative impact of their self-criticism after the first 12 sessions. As noted above, over time participants addressed the fear of changing their hostile self-criticism and became able to notice it and subsequently shift to a more compassionate, self-correcting, self -reassuring and self-encouraging orientation to setbacks and difficulties and change their relationship to self-criticism. In the latter focus groups, participants reflected on their typical triggers for hostile self-criticism and how they could now deal with it and with setbacks in a more compassionate way.

What emerged from a comparison of the three focus groups was how important changes occurred through time in a number of different domains of functioning. Although the extensions were not planned, they enabled the therapy to go over a longer period. Participants began with a relatively ‘intellectual’ understanding of the concepts and their implications, but with extended practise and repetition they gradually became more embodied and ‘lived’ (see also Matos et al., 2018). By focus group 3, one participant explained “*so, what I describe as doing in the group, I have actually*… *I do in the real world as well now, quite easily. And the way I do it just by not making a big deal out of it, not expecting too much, not expecting magical things, but just getting clear in the mind”* (Brian). One participant also highlighted that the exercises have enabled them to access “feeling” states and be able to identify emotional content. Another participant described the process as “*a little bit subconscious*, *you almost don’t think that it made much of a difference, you just find yourself doing it”* (Megan). Awareness of their own minds, and seeing them as changing brain states, facilitated the potential to choose the sort of mind they wanted to cultivate. This insight offers clear implications for therapies that are seeking to change physiological systems: “bodies and brains may take time to change” (Cozolino, 2017; Porges and Dana, 2018).

#### Going Forward

Some clients also felt that this approach would be helpful in many different arenas. For example, Megan noted *“I think it* [compassion] *would do the world of good. I don’t believe that you even necessarily need to have a massive experience of mental health services to benefit from this. I mean, I’d like to see compassionate focused therapy in church halls everywhere. Because people that are under stress, people are under family pressures, bereavement, it works, it’s an all size fits all therapy”.* Participants also made suggestions in terms of what would be helpful in the future. Some suggested organising annual meet ups for continuing support and reminders of the material covered. One way that this could be done is by giving clients workbooks that collate the materials, such as psychoeducation and practices that were given week on week, so they are able to refer back to them in a single document and as a record of their process. This could also be stored on a webpage for clients to access with links to other useful information for them.

### Reflections

As noted in the introduction and Figure 3, psychotherapy seeks to do many things including helping clients become more aware of the nature of their mind, be able to differentiate different functions and processes, be able to tolerate, integrate and transform their minds and self-identities. For the most part, participants reflected on these different processes noticing they had a new awareness of the nature of the mind, were more able to differentiate emotions and change their relationship to their experience of bipolar disorder partly re-orientating themselves to a compassion focus. Many acknowledged being orientated to a more compassionate focused way of living was ‘transformative’ for them. We started the study as partly proof of principle but what this group of participants (and indeed other groups) have indicated is that these therapies can stimulate trauma, and that the ability to work with trauma within the group could be built in to longer forms of the group therapy (Lawrence and Lee, 2014; Lee, 2022). Secondly, we are not necessarily recommending 12-session therapies. We chose 12 to first gain a sense of process feasibility and acceptability, but studies like this raise key questions about how we see the process of change. If we are genuinely interested in biopsychosocial approaches to mental health difficulties (Cozolino, 2017; Schore, 2019; Porges, 2021a,b) then we have to appreciate that with neuroplasticity the process of changing psychophysiological systems can take time to embed and embody and we need considerably more research on these timeframes. In addition, it may take time for people to change their behaviour and then gradually change the pattern of social relationships so that they may be able to become able to co-create more supportive relationships.

## Limitations and Conclusion

Similar to previous studies with patients with a diagnosis of bipolar disorder (Howells et al., 2014; Levy, 2014; Gruber et al., 2015), the current study is limited by a small sample size. The sample size was limited to 10 as the aim of the study was to assess the feasibility and acceptability and was therefore not powered to test clinical effectiveness. Nonetheless, our study provides evidence that clients found the approach understandable and helpful. In addition, there is promising evidence of increases in HRV and positive qualitative data. CFT is one of an increasing number of therapies highlighting the importance of a biopsychosocial approach to psychotherapy, not only as an outcome, but as a process variable. Clearly, further research is needed including to assess the degree to which the therapy can influence the processes that it targets and hence demonstrate clinical effectiveness of CFT in a larger sample of patients with a diagnosis of bipolar disorder.

## Data Availability Statement

The raw data supporting the conclusions of this article will be made available by the authors, without undue reservation.

## Ethics Statement

The studies involving human participants were reviewed and approved by London Central NRES Committee (REC ref 18/LO/1234 IRAS 248283). The patients/participants provided their written informed consent to participate in this study.

## Author Contributions

PG contributed to the study conception. PG, JB, and KM study design and coordination. EN and KL study coordination. AR and AH therapists. FB conducted focus group. PG, JB, KM, JR, PM, HG, NP, SC, and DG data analysis. All authors contributed to the article and approved the submitted version.

## Conflict of Interest

The authors declare that the research was conducted in the absence of any commercial or financial relationships that could be construed as a potential conflict of interest.

## Publisher’s Note

All claims expressed in this article are solely those of the authors and do not necessarily represent those of their affiliated organizations, or those of the publisher, the editors and the reviewers. Any product that may be evaluated in this article, or claim that may be made by its manufacturer, is not guaranteed or endorsed by the publisher.
