# Supplementary material for: Compassion Focused Group Therapy for People With a Diagnosis of Bipolar Affective Disorder: A Feasibility Study
Source: Front Psychol. 2022 Jul 20;13:841932. doi: 10.3389/fpsyg.2022.841932 (PMC9347420; doi:10.3389/fpsyg.2022.841932)
Supplement: Supplementary file 1 [file Data_Sheet_1.pdf]

THE Compassionate Mind FOUNDATION

# Compassion Focused Group Therapy: An Outline of 12 Modules

Gilbert, et al. (2022) Compassion Focused Group Therapy for People with a Diagnosis of Bipolar Affective Disorder: A Feasibility Study. Frontiers in Psychology, 13:841932. doi: 10.3389/fpsyg.2022.841932

The content of this short guideline was developed by Prof Paul Gilbert OBE in collaboration with Drs James Kirby, Nicola Petrocchi and colleagues

# Compassion Focused Group Therapy: An Outline of 12 Modules

**This guideline formed the basis of the modules adapted for**
**Gilbert, et al. (2022) Compassion Focused Group Therapy for People with a Diagnosis of Bipolar Affective Disorder: A Feasibility Study*. Frontiers in Psychology,* 13: 841932.**
**doi: 10.3389/fpsyg.2022.841932**

## Introduction

This brief manual is designed to give an overview of the processes covered in the study, but also offers some details for working with CFT groups in general. Ideally therapists will have experience of group therapy because the relationships between clients are crucial to the therapeutic process. Understanding the complexities of these relationships is important (See Chapters 14 -16 by CFT group therapists in Gilbert & Simos, 2022). Ideally, therapists will be skilled at group focused socratic dialoguing to support guided discovery and to guide compassionate practises.

Like CBT, CFT therapists need particular materials. CFT uses flip charts to write up discussions from the group or sometimes use a laptop and a projector. These discussions are written up and given to clients on subsequent weeks. Moreover, it is useful to have take-away handouts of the basic definition of compassion, diagrams of the three functions (3 circle model) of emotion and other processes, and various thought forms. It can also be useful for clients to be offered personal journals where they keep notes of each session and their reflections on any personal practise that they do. This can be in the form of printed worksheets in a folder/binder with headings such as *Key themes of therapy session X, my reflections and thoughts, the compassion behaviours I will try*. Some therapists and clients may wish to use other headings. the main premise of keeping a journal is to collate materials which they can discuss in the group and keep for months if not years, which can be very useful for some clients. Not all clients will utilise it, however, the therapist should encourage it. Sometimes clients may prefer to record themselves electronically such as on their mobile phones or computers etc rather than, or as well as, writing things down.

Ideally therapists record their practises, such as soothing rhythm breathing and guided imagery, that can be sent to clients or guide them to current recordings and scripts (please

© Prof Paul Gilbert (2022)

see Compassionate Mind Foundation website resources section). Many clients report that they like to use the ones they have learnt themselves within the therapy.

© Prof Paul Gilbert (2022)

# MODULE 1

## Introductions, Setting Working Agreements, Sharing Narratives, and Basic Concepts

**Beginnings** Therapists introduce themselves, invite clients to introduce themselves and then generate discussion to set ground 'agreements on process, respect and safety.' This is followed by exploration of their hopes and fears for the group. Time is spent narrating and sharing their stories with each other. The therapists also introduce a mantra for the group to mindfully set the compassionate intention of being "helpful not harmful" to self and others. This mantra is frequently used in starting subsequent sessions.

Therapists introduce the idea that the group seeks to provide a secure base for encouragement and support and also a safe haven for helping us with our emotions. They explore basic *agreements* for the functioning of the group to create safeness. Issues of confidentiality, respectfulness, safeness and mutual support are emphasised. In addition, the group discusses that therapy can at times be difficult and distressing, and while individuals can take time out, they should not leave the group without communicating this with the therapist. The group should also discuss how they would like to work with some individuals who become over domineering or submissive and withdrawn. Helping people think about these processes can help them to 'own their group' and to see the efforts that are being made to create a secure base and safe haven between all.

Therapists give opportunities for a brief discussion about active listening and how they can do that. They also give a brief insight into socratic dialogues and that sometimes they may want to ask questions of each other so they can understand the thoughts feelings and motives of each other and how we can do that. Learning 'to take an interest in the minds of others' can take time with some groups. To guide this, a therapist may often want to invite people to consider "would you like to know more about what X was thinking or feeling, and how might we like to ask and discover?" The therapist stimulates clients' abilities for therapeutic interactions throughout the course of the group work.

**Exploring Compassion** Here therapists explore the group's ideas of coming to a compassion focused therapy group and their current concepts of compassion. Given there *are now*

© Prof Paul Gilbert (2022)

*different definitions of compassion, some of which are not so helpful for therapy,* clients are guided to the definition using an example of how they would help a friend who was frightened of going to hospital (for details see Gilbert & Simos, 2022, Chapter 7, pages 243-244). Therapists elicit the awareness that they would be sensitive to their friends' distress and would then try to work out how to be helpful. This provides the basis for their understanding of the algorithm (*if* A *then* do B) of compassion and hence the definition of compassion as (A) *sensitivity to suffering in self and others with (B) a commitment to try to alleviate and prevent it*. Compassion is different from caring, as seen in other animals, because it requires us to use our *knowing awareness* and different forms of intelligence such as empathy, reasoning and mindfulness which support the two key features of compassion of courage and wisdom (see Gilbert & Simos Chapter 2, pages 65-66). This definition of compassion is clearly distinguished from kindness, love and other prosocial concepts. Those are ways of being compassionate but can be off putting to some clients in the first instance, whereas recognising that they can develop *courage and wisdom to address suffering* tends to be easier to take on board. Clients also discuss how different skills and behaviours are involved in different types of compassion. For example, the courage and wisdom to be a firefighter and rescue people is very different to the courage and wisdom to help the homeless or be a counsellor. Hence, compassion (as defined above) is not one process but is specific to the nature of the problem. For self-compassion, the courage, wisdom and commitment to help us confront say problems of obesity or fear of death might be different to that of depression or trauma. Clients are invited to consider the areas where they may benefit from compassion, but also potential problems (called fears, blocks and resistances, see Gilbert & Simos, 2022, Chapter 6, pages 221-237) with applying compassion to themselves.

**Flows of compassion** Like other motives and emotions, there is a flow of compassion - that is we can have compassion for oneself, openness and responsiveness to the compassion from others, and generate compassion for others. Clients discuss these and note that some of the flows are easier than others. Group therapy can be particularly helpful for working with all the flows of compassion (see Bates, 2005 and Chapter 15 by Griner, et al., in Gilbert & Simos 2022).

© Prof Paul Gilbert (2022)

**Helping the body to support the mind** Therapists discuss the concept of 'helping the body to support the mind' and in particular how we can use embodied practices, yoga, movement and breathing patterns to slow and ground us when distressed. Hence, clients are introduced to the first concepts of the use of posture and Soothing Rhythm Breathing Practice (SRB) and 'body awareness' (e.g., note the sense of grounding with the breathing). Throughout all subsequent modules, clients practice these skills and discuss how the practices relate to them personally and how they can use them. Clients are invited to explore the internet to try out different breathing patterns to find what might best suit them. Therapists can give a very short and basic overview of the autonomic nervous system and how breath training can affect it (see Chapter 10 by Petrocchi, et al., in Gilbert & Simos, 2022). Depending on the group, if they are taking time to settle into their relationships and familiarising themselves with the process of group therapy, this may come in later sessions too. As clients are practising these together, this can be both helpful but also individuals may compare themselves to others and judge themselves negatively. This should be noted as a potential issue with consideration of how to deal with it.

© Prof Paul Gilbert (2022)

# MODULE 2
## Psychoeducation and the Evolutionary Model

**The Evolved Tricky Brain and Emotion Systems** Psychoeducation is an important part of CFT. Clients are introduced to the evolutionary reality that all living things are built by their genes with a set of dispositions for survival and reproduction. No living thing chose to be here or in the form that it is. Hence, we have brains and bodies built for us not by us with dispositions for a range of desires and emotions (see Gilbert & Simos, 2022 Chapter 6, pages 249-254). Therapists explain the social patterning of the mind and sense of self, that if they (the therapist) had been kidnapped as a three-day old baby, they would be very different to how they are today. They touch on differences in epigenetics, brains, bodies, minds, beliefs and values etc. Hence, we do not choose the versions that we are. This highlights a core theme of CFT which is that much of what goes on in our *brains is not our fault*, but it is our responsibility to try to understand our evolved programmes and activated algorithms so that we may behave helpfully not harmfully. For example, we may note a shift into the anger algorithm but not act out aggressively. Clients are then invited to spend a few moments in silence just reflecting on this reality. The therapist can use very gentle voice tones to highlight the fact that *we never chose the minds we have, much of what goes on inside them is not our fault*. Therapists outline the nature of social mentality (see figure 2 of the paper). Clients are invited to consider and discuss personally relevant experiences, which helps them connect to the motivation switching within them. Now, but also throughout the group modules, there is a focus on what it might be like if they could notice and then shift motives to mindful compassion.

**Evolutionary functional analysis of emotion** CFT helps people consider the *functions* and *forms* of their psychological processes. For example, what is *the function* of my anger, anxiety, sexual feelings or compassion and what form do they take, how do they manifest in my thoughts, body, behaviour and so on? CFT clusters emotions in terms of their support of three major life tasks with three evolved main functions: (1) to detect and avoid threats and harms; (2) to detect and engage with seeking and acquiring resources; (3) to detect opportunities for rest and digest. Therapists indicate how the threat system tends to be problematic for people

© Prof Paul Gilbert (2022)

with mental health problems (and in general too) especially the emotions of anger, anxiety, sadness and various numbing and shutdown states. The function of all these is mostly defence and/or adaption to losses. Clients are invited to consider that emotions have different aspects to them. For example, if we are anxious, we will *think* in a particular kind of way, have experiences in the *body*, might *remember* past events, and want *to do* particular things. Generally, thoughts and behaviours centre around the meaning and implication of events and actions. Clients are invited to discuss with each other these different dimensions of an emotion. For example, the typical thoughts they have when they become anxious or angry and the typical behaviours they feel they want to act out. These discussions create a sense of common humanity on the threat system (and de-shame) and highlight how powerful the threat system can be in taking control of our minds. They are then offered a thought experiment of what would happen if they could switch to a compassionate state of mind; how would that change the way they thought, what was going on in their bodies, what they might want to do. If they were anxious or angry, what might happen if they notice that and then, deliberately try to switch to thinking about what would be most helpful to them in that moment of distress. Again, these are offered for discussion between clients. The therapist tries regularly to introduce the idea about the potential to switch and refocus motives and mental states.

Clients are guided to consider inner conflicts. For example, clients can be invited to consider that different emotions can be activated at the same time causing complex conflicts between emotions. You can ask clients "*what combination of emotions they felt when thinking about starting compassion focused therapy and meeting new people or perhaps coming today*?" The therapist uses a lot of socratic dialoguing to elicit the complex variety of emotions, which may range from anxiety, doubt, interest, hope etc. All these can be written up on your flip chart because the more clients see these processes becoming manifest on a flip chart the clearer it is for them, and this helps differentiation function. We can learn to become more aware of our multi-minds by slowing down and deliberately asking ourselves about what different emotions we might be feeling, and consider the function, rather than just 'feeling and acting' on them. Hence, the therapist is helping clients *differentiate* different processes of mind and

© Prof Paul Gilbert (2022)

also label them, which is a powerful mindfulness intervention. The above helps the processes outlined in figure 3 in the paper.

In addition, people with this diagnosis can experience elevated drive for resources, excitement and exploration but can switch into shut down, withdrawal and loss of positive emotion. Clients explore ways of understanding their emotions using this framework. Importantly, the group discusses this way of thinking about mind switching as linked to these evolved motivational systems. The ability to become mindful, stand back and observe one's *social rank system switching emotions on and off* can be helpful. This is accompanied by deliberate attempts to switch on their compassion motivational system which can help (even if minimally) to trigger a different brain state. The therapy helps clients understand and become more attentive (mindfully mind aware) of how these systems are working within them at any point in time, their activators/facilitators and deactivators/inhibitors. Clients are invited to share stories of how these emotion regulation systems became 'patterned' as a result of life experiences. Hence, clients can explore how the threat system has become sensitized to particular triggers or the 'drives' they experience when they become hypomanic.

© Prof Paul Gilbert (2022)

# MODULE 3

## The Nature of Caring Behaviour, Secure Base, and Safe Haven

**Attachment:** CFT is informed by attachment theory and the research on caring and prosocial behaviour in general (See Gilbert & Simos 2022, Chapter 3). These are discussed briefly with clients, particularly how early attachment, caring or bullying relationships can affect us later in life. As part of this, therapists introduce the concepts and processes of a secure base and safe haven. A secure base provides children and adults with encouragement, validation, and empathic support, while functions of a safe haven are to help settle, feel safe, soothe and ground us. Clients discuss how the group itself can be a source of a secure base with encouragement, understanding, validation, mentalisation and a safe haven with soothing and grounding. When developing our compassionate minds these are the kinds of competencies, we are seeking to develop so that we can have the capacity for self-validation, mentalisation, encouragement, reassurance and soothing etc.

**Safety and Safeness** CFT distinguishes between these two by suggesting that safety is primarily regulated through the threat system (e.g., amygdala and sympathetic arousal) and is attentive to the presence, absence or controllability of threat stimuli. These can be external but also internal (e.g., a sense of threat from flashbacks or intrusive thoughts or impulses). Safety behaviours therefore can be ones of avoidance, aggression, and helpful or unhelpful ways of trying to cope and regulate threat to reduce its impact. A sense of safeness can obviously be linked to a sense of the absence of threat but in addition it is linked to the perceived availability of helpful others (as in attachment theory). Hence, rather than trying to cope with the threat directly and alone, when individuals feel safe, they are (also) able to turn to others for support. For example, young children are not able to provide for themselves in any meaningful way, nor are they able to regulate their emotions but require the parent/carer to do that for them. This means that both positive affect systems and threat regulation systems are influenced through the care system. The awareness that others can be helpful, and soothing creates a sense of social safeness. Considerable evidence suggests that when we face a threat alone, we physiologically respond quite differently than when in the context of supportive and caring others. Cues of social safeness tend to work through the

© Prof Paul Gilbert (2022)

parasympathetic system and hormones such as oxytocin. By practicing techniques that can help develop the vagus nerve this can help balance the autonomic systems so that emotion tolerance becomes easier. Clients may come to understand that this process is difficult for them if they have had difficult backgrounds and therefore, they may need to learn how to stimulate it within themselves.

**Imagery:** Therapists clarify what imagery is. For example, therapists ask clients playfully what an elephant is, what colour is it? How many legs does it have? What's a bicycle? They then ask clients how they know these things, what comes into their mind. Imagery interventions are used regularly in therapy, so it is important to address any misunderstandings. Therapists indicate imagery as sensory awareness, not necessarily clear visual depictions, and the value of images 'felt in the body'. Therapists are also aware that some clients will struggle with imagery (aphantasia) but may be able to use different sensory modalities.

**Safe place imagery** Therapists introduce the concepts of safeness and safety and use imagery to distinguish the two. They invite clients to create a safe place and explore the experience of safeness, imagining the place welcoming them and feeling connected to the place. Clients are invited to explore the differences between being in a place that is non-threatening (place of safety) versus a "welcoming and taking pleasure in the presence of the self" (place of safeness). For some clients a sense of being "welcomed and belonging" can be difficult and new for them and hence an important practice. Safe place can be where we simply feel safe and relatively calm and quiet, perhaps being in a beautiful garden, walking by a gentle stream, watching a soft surf. However, some clients prefer a much more active aspect to safeness which involves play and the drive system. Clients therefore are also invited to explore the importance of "freedom to play" and being spontaneous and creative when they feel safe. Safe place imagery can be used for grounding and exploration and "having fun." The concept of "safe play," which evidence suggests was key in hunter-gatherer societies, is important in CFT.

© Prof Paul Gilbert (2022)

# MODULE 4
## Attention and Mindfulness

**Attention and mindfulness** (See Gilbert & Simos, 2022 Chapter 8). Many therapies now regard mind awareness and mindfulness as central to therapy. As this group had already been introduced to mindfulness during the Mood on Track programme that the clinic provides, therapists recapped on some of the basic concepts linked to attention processes. Generally, clients are guided to note how the focus of attention can be recognized and then moved (for examples, see Gilbert & Simos Chapter 8). Clients are given a brief overview of what mindfulness is and isn't, with some simple examples. Cultivating *habits* for mindfulness and staying in the present moment and becoming sensitive to sensory information can be suggested for behaviours such as: breathing, walking, eating, showering and dressing etc. We can become mindful to our senses and learn to hold them in mind. Forms of mindfulness can also be useful for grounding. This is not to engage in avoidance, but to bring balance to one's mind. Such practices help to settle the mind, develop an observing mind that becomes less fused with thoughts, worries and plans.

Clients are invited to see body and attention as a two-way street. For example, when we have physiological shifts in mood or emotion (as in mood regulation problems) this changes what we attend to. We can become aware that the body, feelings, thoughts and attention are all connected, but we can also stand back from them and become mindful of these brain state patterns being created in our minds without being overly 'fused' with them. Mindful guidance invites observation of components of brain states such as, thoughts, emotions, motives, and behavioural impulses associated with any particular brain state. Where possible clients can move to the next issue of what is the function of these and what would be helpful ways of being with these.

Clients are reminded that compassion is an S-R algorithm and while it's important to be mindful and attentive to the (stimulus) nature of suffering, it is also important to shift attention to focus on "the response" of what can be helpful. Therapists highlight that what goes out of mind (e.g., helpful thoughts or memories or past coping) can be as important as

© Prof Paul Gilbert (2022)

what comes into mind (e.g., fears and worries). Hence the values of directing attention to what might be helpful.

**Loops and Ruminations** Clients are invited to discuss the way in which patterns of attending and thinking can become patterns of ruminations and create recurring unhelpful loops. Clients are guided through some basic cognitive therapy skills for ‘helpful thinking’ and ‘helpful behavioural planning’. Mindfulness can help clients begin to become more aware of their patterns of rumination and how to notice, slow down, slow the breathing and refocus the mind. During all phase’s clients share ideas which are written up on a flip chart for discussion, particularly of how easy or difficult it will be to use these interventions and how to address difficulties.

**Group Dynamics:** It was clear that many individuals in this group were very keen to express their own views and direct discussions. The therapists discussed the group dynamic of competing for space and practiced mindful awareness of this and switching to the compassionate orientation of the flows of compassion. At times the therapist can invite clients to *playfully* notice what it feels like to have an urgency to speak or to share.

Clients share and discuss their own stories around these themes during the therapy. Mindfulness is also used to help us track our intentions and how to tune our attention. Hence, if we have the intention to live compassionately and helpfully then we can become mindful when we are having experiences that pull us away from that intention and could be harmful to ourselves or others. Mindfulness can therefore be compared to the driver of the car who is paying attention to what is happening and keeping the car moving forward safely.

© Prof Paul Gilbert (2022)

# MODULE 5
## Building the Compassionate Mind and Self

**Building the compassionate mind and self:** As compassion motivation has such a crucial impact on the organization of multiple psychophysiological processes and brain states, helping clients understand what it is and how to create and practice it is essential (see Gilbert & Simos, 2022 Chapter 8). The care-compassionate social mentality becomes the lens through which other interventions are enacted. Hence, the therapy returns to the basic definition of compassion and opens further explorations into what a compassionate mind and a compassionate sense of self would be like. Clarity on the mind organising properties of compassion is essential. Clients are then invited to generate their own ideas of their compassionate self (e.g., being a good listener, having patience, being friendly, caring, tolerant, supportive etc.). These are written up on the flip chart as examples of their intuitive wisdom about the qualities of compassion, how to cultivate them and the value of doing that. Clients are then invited to think about the psychoeducation of tricky brain and the wisdom that we all just find ourselves here with 'mind-programmes' that we did not choose. The therapy then revisits these challenges of our minds and how compassion motivation can help develop courage and wisdom to work with them. So, for example, if a client chooses the idea of patience, then 'how would patience help them to become courageous and wise in facing their difficulties'? Clients are understanding how they can tap into one aspect of the mind (e.g., compassion) to help them with other aspects of mind (e.g., threat processing).

**Body and mind:** Clients are invited to go through a process whereby they engage in adopting an open posture, practiced soothing rhythm breathing (diaphragm breathing around four breaths per minute), generated friendly face and intent, focused on compassionate voice tones and then imagine becoming their version of a compassionate self they have conceptualized. Therapists also introduce various acting techniques such as inviting people to walk around the room as the compassionate self and contrast that mind state with (say) an anxious self. The therapist is enabling clients to develop the sense that brain states are not set in concrete but can be changed in subtle ways through focusing and practice. They also help the process of diffusing, decentering and dis-identification with particular brain states.

© Prof Paul Gilbert (2022)

Acting techniques such as ‘acting as if’ and ‘acting on’ and acting out’ are used frequently in CFT as aids to exploration and guided discovery, body awareness and exploring new possibilities for the self.

**Compassion focusing:** Clients are invited to think about and discuss a small life problem in the emotion of the problem. They note how they think and feel about the problem in that state. They are then invited to do a compassionate mind state induction associated with breathing, grounding compassion, wisdom and focusing compassion qualities (intention and process). Then they consider the problem through their compassionate mind state. Such practices help clients to see brain states can be changed, but also when they try to create a compassion brain state, they can have access to different ways of feeling and thinking about a problem and find their own internal wisdom and courage. In some groups you can invite people to do this exercise in pairs. As in all group therapy interventions, therapists will be aware that clients can experience these differently and to support those who struggle.

**Cognitive behavioural supports:** Clients are invited to explore a variety of cognitive behavioural ways of being helpful such as identifying styles of reasoning and considering if there are more helpful ways. The clients in this cohort had already been part of a cognitive therapy program and so were familiar with this. This group added the importance of creating a particular mind state and compassionate motivation and focus to the cognitive dimensions.

**Letter writing:** Clients are guided into using compassionate letter writing. In this practice clients activate a compassionate mind state (via the breathing practices, focused attention, evolution wisdom and orientation) and then write a letter to oneself about certain life difficulties. Sometimes clients are invited to read out their letters to the group and sometimes other members of the group can read each other’s letters, ensuring that they create a compassionate and kind voice tone when they do it.

Throughout, clients share and discuss their own stories and experiences around these themes during the therapy.

© Prof Paul Gilbert (2022)

# MODULE 6

## Openness to Compassion and Compassionate Image

**Building an inner compassion image:** Clients are advised that we often think in interactional and dialogical terms such as in our sexual or angry fantasies. We also fantasize what others may be thinking about us. Clients are invited to imagine dialoguing and relating to a compassionate other mind. Keeping in mind the nature of imagery and its often vagueness, they can nevertheless consider what qualities they would like that other mind to have (e.g. appearance, manner, voice tone, empathy); how they would like that (other) compassionate mind to relate to them, and how they would like to relate to that (other imagined) compassionate mind. It helps to use various examples of how our imagination stimulates our body; for example, notice how different the body is if we imagine winning the lottery than if we imagine having an argument. In some groups, therapists could also use the sexual imagery example because it's such a powerful example of *imagery creating physiological change*. Compassionate imagery can change brain and body!

Clients are reminded of some of the functions that can underpin a compassionate mind such as it could offer secure base in the sense of being understanding encouraging, validating and supportive and also can act as an internal safe haven where the interactional dialogue creates a sense of grounding and soothing. Clients can practice scenarios of both, keeping in mind that clients might not have any clear visual images but just impressions and a ‘sense’ of an interaction. It is important that clients are able to create inner experiences that are genuinely experienced (even minimally) as helpful rather than ones they think should be helpful, but actually are not. Clients also explore their fears, blocks and resistances to being able to imagine being cared about in certain ways. These are shared with the group with ways of moving forward step by step with them. Some people find it difficult to imagine another human being/mind as their compassionate image but can imagine a particular animal representation. The therapist helps clients recognise that this process can take time, they can have more than one image as suits them. Clients are guided to recognize that when we imagine certain types of interpersonal interactions these can stimulate particular algorithms

© Prof Paul Gilbert (2022)

and physiological systems in the mind. This is no different from how we use imagery in general (e.g., sexual or planning exciting activities) to stimulate brain states.

**Openness to the group:** The focus of openness to compassion is explored in relationship to the group and how people experience being helpful to others and the desire for others to be open to that helpfulness rather than push it away. As part of experiencing compassion from others, the therapist can sometimes offer gratitude memory practices and to distinguish between joyful appreciation versus guilt or obligation acceptance.

**Compassion to others:** Clients discuss how they feel when they can be helpful (e.g., validating, sharing, empathic, encouraging, supporting) to others and others clearly benefit from that helpfulness. This opens up discussion that humans want to feel valued and able to connect and make a contribution. Sometimes we can feel depressed if we feel we do not have anything of value or people do not value us. Then if we become hypomanic, we can feel very valuable. Clients explore the difference between considered, mentalised helpfulness and impulsive 'hypomanic impulsiveness'. The therapist constantly returns to the 'process of flow' not only the ability to try to help others but also to be open to the help from others. Sometimes this raises issues about feeling others have let one down and the problems of mistrusting others and their intentions. These can become powerful discussions within a group and how to 'compassionately recover' abilities to engage the flows of compassion.

Clients shared and discussed their own stories around these themes during the therapy.

© Prof Paul Gilbert (2022)

# MODULE 7
## Multiple Selves and Mixed Brain States

**Multiple Selves** is one of CFT's set pieces (Gilbert & Simos, 2022, Chapter 9). It has many functions. For example, it helps with developing people's mind awareness, their ability to differentiate the different aspects of different emotions, and with insight comes increased tolerance and flexibility (see figure 3 in the paper). When we engage a compassionate mind to our emotions this facilitates integration (see figure 3). This practice also helps clients recognise that we rarely experience single motives, desires, thoughts or emotions but complex changing patterns of each. For example, in relationship to trauma or a life difficulty, such as being diagnosed with cancer, or working with a mental health problem, we very often experience what in CFT we call the "big three" of: anger, anxiety and sadness/grief. We can be angry at what happened and why, we can be anxious about the implications and future and we can be sad at the losses it imposes on us and what it might have robbed us of to help clients recognise we can have these multiple emotions, motives and beliefs to the same event. The therapist guides clients through an example that involves complex conflicts of emotions, motives and thoughts.

A common example CFT uses, that typically involves the elicitation of a range of different motives, emotions, thoughts and behavioural impulses, is to recall having an argument with somebody they care about and then explore in detail the big three emotions of: anger, anxiety and sadness (see Gilbert & Simos 2022, Chapter 9, pages 314-327. Clients are invited to focus on one emotion at a time and then explore that emotion in detail in terms of its attention, motivation, thinking, body activation, behavioural impulses, memories and ways of settling the emotion. Clients explore how these emotions (anger, anxiety and sadness/grief) have their own structure of attending, thinking, behaving and so forth, which can be very different from each other and can be in conflict with each other. Clients can be invited to explore "*what does your angry self think and feel about anxious self; what does anxious self think and feel about angry self* etc. in any combination including with compassion itself; for example, "angry self" can dislike and try to suppress "anxious self" or "sad self" and vice versa. Deepening

© Prof Paul Gilbert (2022)

insight into the complexity, variation and relationship between such emotions supports mind awareness, mindfulness, differentiation and integration.

Clients are then invited to try to activate their compassionate mind-state using the compassionate mind induction of posture, breathing and focusing intention and wisdom, for a minute or two. They then consider the argument through the lens of the compassionate mind. This commonly generates new ways of thinking about the argument. However, if this has not spontaneously occurred, the therapist can guide clients to consider mentalising the argument and the helpful ways of moving forward. In addition, whereas emotions in the threat system (anger, anxiety and sadness) tend to be in conflict with each other, when clients bring a compassionate mind state to those emotions, they recognise that their compassionate mind can empathize with each of the threat emotions, but not allow them to control the mind. Throughout the practice the therapist writes up their responses on a flip chart.

Clients are guided to consider the many different ways in which things happen throughout life and the combinations of difficult emotions when dealing with them.  The therapist invites clients to think of their own real-life examples where they have had different emotions to the same issue, and the value of being able to be aware of those different emotions and perhaps focus on them individually. The therapist invites the group to think about how they might practice using these skills (of awareness, differentiation, tolerance and integration, (see figure 3 in the paper) by learning to slow down, focus, explore for possible multiple streams of thought or emotions and switch to a compassionate mind. Learning to identify and label separate processes helps mindfulness and decentering from them. Clients are guided into how they can use these techniques of ‘noticing multiplicity and then shifting to a compassionate mind’ in many different aspects of life including coping with dilemmas, and life challenges.

Clients share and discuss their own stories around these explorations and efforts during the therapy.

© Prof Paul Gilbert (2022)

# MODULE 8
## Self-Criticism

**Self-criticism** (see Gilbert & Simos Chapter 5 for details on how to conceptualise and engage in a range of therapeutic interventions with different functions and forms of self-criticism). Harsh forms of self-criticism are usually associated with hostile emotions of anger and contempt. These are common to many psychological difficulties. CFT utilises an evolutionary approach that links self-criticism to the regulating processes in dominant-subordinate hierarchies, whereby those individuals threatened by dominant others have to engage subordinate and submissive behaviours. Many harmful forms of self-criticism are therefore linked to *threat and social rank-based processes* of social put down and shaming. It is important to distinguish *hostile and shaming* forms of self-criticism from simple processes of acknowledging errors of behaviour. Again, CFT makes key distinctions between the motives and emotions that generate self-criticism. Harmful self-criticism is rank, and threat based and undermines confidence, whereas supportive self-criticism is friendly, caring, encouraging, correcting and builds confidence and desire to 'learn more'.

A key step is to recognise that some forms of self-criticism hide anger towards others in the classic psychodynamic and behavioural sense. For example, children who are regularly threatened and criticised will often take the blame and engage submissive and apologetic behaviour towards a parent in order to avoid being hurt. They are not in a position to defend themselves with aggression or counter threat. In therapy, they may be anxious of anger or assertiveness. Self-criticism can be linked to internalising a persecuting other's judgment of the self. This is discussed in the therapy and how to address it.

A second process seeks to help clients distinguish between feelings of frustration and disappointment in themselves versus potential self-critical responses to frustration and disappointment. Therapists explore the ability to cope with the different emotions of frustration, disappointment and setbacks without becoming harshly self-critical. Clients learn that self-criticism can often block deeper feelings about a frustration, disappointment or setbacks (e.g., the big three of anger, anxiety and sadness) and learning to tolerate those

© Prof Paul Gilbert (2022)

feelings in themselves or work through them, without self-criticism getting in the way, is helpful.

While hostile forms of self-criticism are common in mental health difficulties, the therapist helps clients recognise that self-monitoring and self-blaming in themselves may not be problematic as they can be simply be about causality. It is the emotions of anger, contempt or even hatred of self-attacking, that create hostile forms of verbal and emotional attacks, that is harmful. As a guided discovery, clients complete a 'Functional Analysis' experiential exercise. (This is described in detail in Gilbert & Simos, 2022, Chapter 5, pages 187-201). Clients are advised to pull out anytime if they find it too difficult because guided discovery is not designed to be overwhelming but revealing. In this exercise clients are invited to think about their fears of giving up attacking/shaming/self-criticism. Usually, people fear they will lose focus, ambition or drive, becoming lazy or unacceptable in some way with a deeper archetypal fear that this will lead to social diminishment, devaluation and possible rejection. In order to stop them becoming like that, and risking those outcomes, they have developed beliefs that they need the critical process to keep them from "slipping." Part of this guided discovery is to show them this is not the case, that hostile self-criticism tends to be undermining and harmful not helpful, and that compassionate self-correction, support, guidance and encouragement will help them much better.

The actual procedure involves inviting clients to a "guided discovery" to explore their self-critical process in detail. As a group exercise, the therapist writes up all of the responses to the guided discovery on the flip chart.  First clients are invited to bring to mind something they are critical about for about 20 seconds, just enough to engage the sense of self criticism. When they have engaged with those feelings, then they are asked to imagine the 'critic' outside of themselves, as if it became an entity, look at it and listen to it. Then consider: (1) what might their inner critic look like, what sort of colours, what forms might it take  (2) if it could say whatever it wanted about you and to you, what would it say (e.g., you are lazy, stupid, no good, inferior etc), (3) what  feelings does the critical part express to the self (e.g., anger and contempt), and finally (4) what would the critical part of self want to do to the self (e.g., kick, shout, push the self into action). Clients are then asked to consider how they're feeling after experiencing this 'guided discovery'. They often recognise they feel low in

© Prof Paul Gilbert (2022)

confidence and “beaten down and discouraged.” Sharing and seeing these responses written up helps clients feel less alone with their critic and view it is a common human problem because of the way our minds are.

Clients commonly discover that the critical process is hostile and undermining. Clients are then guided to the alternative which is called compassionate self-correction, reassurance and guidance (secure base). Clients are invited to spend a short time with a compassionate mind induction using the breathing and wise focusing and then to focus on the motives of this mind. Often the compassionate self wants the self to be at their best, to be successful and to be helpful to self and others and find meaning and joy in life. The motive for compassionate self-monitoring is to improve, guide, encourage, empathize (with setbacks, frustration or disappointment), be supportive and act as a secure base and safe haven.

Clients are invited to go through the same four processes but this time with a focus on the compassionate mind or compassionate image; what it might look like, what it would say to the self, what it feels for the self, what it wants to do to help the self. It is important that a therapist ensures the client generates genuinely helpful thoughts and textures, not ones they think should be helpful or sound compassionate, but do not impact the client. Hence, the therapist will ask “when you think like that over here, that sort of voice in your mind, how do you feel in your body? Although you think it should be helpful, is it? After exploring these dimensions clients are invited to consider how they now feel. Clients are then invited to explore the differences between hostile self-criticism and compassionate self-correction, guidance and encouragement. There are many aspects to this which is covered in the chapter mentioned above.

To develop insight into the functions and nature of self-criticism, clients are invited to think about what sits behind their self-criticism. For this, clients are invited to imagine becoming the compassionate self and then viewing/imagining the critical self in front of them engaging in criticism. The participant is then invited to imagine moving around the back of the critic (a kind of ‘wizard of OZ’ manoeuvre), and thinking about how it started? What were the wounds or threats that set it into action? The Therapist can explore: what is driving it? What does it fear? What does it need and if it gets what it needs, how will that be? Keep in mind that the therapist is actively writing their responses up on the flip chart and discussing them as they

© Prof Paul Gilbert (2022)

emerge, inviting discussions between clients. In the next stage the therapist invites them to consider how might their compassionate mind be compassionate *to the fears behind the critic* (which are usually archetypal fears of rejection or devaluation). In other words, clients are guided to be compassionate to the fears behind the critic not to the hostile critical process itself. In fact, there is little engagement with the critical part. Clients can be invited to explore the impact of the harsh self-critical process and areas of their lives where they think it has undermined them or generated anxiety and depression rather than generate confidence and joyfulness. This is because self-criticism is rooted in threat processing whereas compassionate self-correction, reassurance and guidance are rooted in the care system. This exercise also allows clients to realise that hostile self-criticism is not simply a misguided form of trying to be helpful because it's coming from the threat system. In CFT, hostile self-criticism is generated by fear, not desires to be helpful.

Clients share and discuss their own stories around these themes during the therapy.

© Prof Paul Gilbert (2022)

# MODULE 9
## Shame and Guilt

**Understanding shame:** Therapists provide psychoeducation on the nature of shame and its link to social competition for social place and social acceptance (see Gilbert & Simos, Chapter 4). CFT distinguishes clearly between internal and external shame. Internal shame and internal shaming relate to how we think about and relate to ourselves. It is therefore a dimension of self-criticism which is covered in the previous module. External shame focuses 'being shamed', on social comparison and fear of being looked down on, devalued, diminished, subordinated, and rejected by others. These are typically the fears that the self-critical process focuses on too. External shame can be associated with mixtures of confusion, a sense of paralysis, anxiety, disgust, and contempt, and occasionally anger and we seek to defend ourselves against belittlement. Therapists discuss why, for humans, social devaluation is a major threat that our brains are sensitive to because we are a species that wants to be connected, chosen, and wanted, seen as desirable and fears isolation and rejection. External shame is commonly linked to the fear of exposure. Therapists explore how we can pick up a sense of shame from early life experiences from bullying, criticism and rejection which can underpin mental health problems. Discussion is on issues of fears of revelation 'shameful experiences' but without pressure to reveal. We can experience shame when we want to reveal shame. These fears are normalised, and clients may choose to discuss shame themes in general rather than personal shame in the first instance and gradually move towards more open discussion. Therapists highlight the challenge of how to engage a compassionate approach to shame. Sometimes this can also relate to the issue of self-forgiveness and therapists can guide clients to what that involves and what it does not.

**Guilt:** Whereas shame evolved through social competition and regulation of social behaviour, guilt evolved from the caring system and links to harm and harm avoidance. Hence, guilt is closely associated with feelings of remorse and sadness rather than ones of anxiety, disgust or contempt. Clients may talk about coping with guilt as different from coping with shame and that guilt like other emotions may require some degree of tolerance. The ability to acknowledge harmful behaviour and process guilt can be important for some people.

Clients discuss their own stories around these themes.

© Prof Paul Gilbert (2022)

# MODULE 10
## Assertiveness, Apologies and Forgiveness

**Conflict:** Therapists raise the issue that because we are biological beings, we, like other animals, often have all kinds of conflicts with each other. These can occur with people we do not like but also in relationships that are important to us. Conflicts can be difficult but also opportunities for growth. Clients discuss how and what types of conflict are particularly difficult for them and whether or not planning to be compassionately assertive would be helpful.

**Motivation**: The group discuss one's orientation in a conflict, whether it is 'to win' or whether it is to cooperate to resolve the conflict as best we can. Our motivation can set us up to engage conflict in different ways. We keep in mind that we all have tricky brains that we did not choose. Clients can be guided through the process of active listening where they listen to what the others say and then repeat it back to them, trying to really understand the issues and concerns in the one we have conflict with. Therapists can revisit issues of how we can use socratic ways to explore the mind the other. Clients may raise all kinds of issues that other people do not listen, or they are not interested, and the therapist guides them through how to maintain one's own position and approach even in the face of such difficulties, for example trying to stay mindful of the flow of one's thoughts and impulses that may pull oneself away from compassion intention. CFT also opens discussion around the three main issues of conflict which are (appropriate) *assertiveness, apology and forgiveness*. The therapist helps clients to recognise that conflicts are to do with two (or more) minds that have their own desires, wishes, motives and fears interacting with each other. Clients also discuss the themes that sometimes relationships are toxic, and one needs to move away from them and the courage and wisdom that can take but also the barriers to being able to do that.

**Assertiveness training:** Conflicts can sometimes be difficult because individuals either move impulsively into anger-attack or they can be fearful of anger and conflict and thereby suppress their concerns, points of view and are overly submissive. Some can submit but be resentful and some sulk (passive aggression). Therapists can explore these different ways in which clients can relate to conflicts and then begin to think about how clients would like to be able

© Prof Paul Gilbert (2022)

to work with conflicts if they are at their compassionate best. Key issues then are how to help people recognise ‘anger’ and to be neither ‘aggressive-threatening’ nor submissively resentful but cultivate the *courage and wisdom of assertiveness*. CFT utilises many standard assertiveness training interventions from other models. Clients explore the four dynamics of assertiveness; (1) to promote one’s own ideas, creativity, individuality; and (2) to defend one’s position, boundaries etc; (3) to accept and take joy from praise (4) able to experience show gratitude and appreciation. Clients consider how issues of shame and guilt can interfere with these aspects of assertiveness and how to take a compassionate approach.

Assertiveness also involves mentalisation skills. Central to compassionate assertiveness is courageous wisdom with wisdom based on empathy for self and other. We warn people away from concepts of aggression or fierceness as ways of dealing with conflict. Clients are given opportunities to practice courageous and wise (mentalising) assertiveness. Simple examples can be practising imagining taking something back to a shop that you do not want or is broken, sometimes helping clients think about parenting skills and providing them information about how to deal with conflicts with children, which the Internet can be excellent resource and helping individuals accept and compromise rather than ‘needing to win.’

**Apologies:** Therapists discuss that in conflict situations and in other areas we can make mistakes that cause suffering to others. This raises the question of how we can genuinely acknowledge that without engaging in shameful hiding or denial and where it is appropriate and helpful to apologise. Apologies can be another dimension of courageous wisdom. Exploring the compassionate aspects of apology can help to counteract negative beliefs about apology and understand how genuine apology is assertive not submissive and builds relationships.

**Forgiveness:** Given the growing literature on forgiveness and the value of forgiveness for self and others, therapists discuss what forgiveness is and isn’t and how it is beneficial to the self and also relationships. The focus of forgiveness is not to dismiss hurt, fear or guilt but to let go of or tone down anger and the desire for vengeance, resentfulness and attacking. The therapist may have a brief discussion about how holding onto resentments can be bad for our health. For example, it keeps the threat system stimulated. However, there is no ‘should’ to forgiveness, only an understanding of the process and whether it is helpful. The therapist

© Prof Paul Gilbert (2022)

should be familiar with the different stages and steps of the forgiveness process that starts with an acknowledgment of hurt and maybe grief or rage rather than a suppression of them.

Clients are also invited to explore how resentful ruminations to themselves or to others can undermine self and relationships. Hence, the importance of self-forgiveness and facing the blocks and resistances to it.

© Prof Paul Gilbert (2022)

## MODULES 11 AND 12
## Reflections and Progress

The last two modules provide opportunities for clients to reflect over their earlier group modules and how they are integrating them into their everyday lives. All psychological processes have facilitators and inhibitors, and clients explore things that can help them and things that might hold them back in the weeks and months ahead and what they can do about it. The main functions of these modules are to enable opportunities for embedding practice and clarity about compassionate living. These modules allow for revisiting key themes, practicing and deepening insights, further group sharing and thinking and preparing for how one wants to live a compassionate life.

© Prof Paul Gilbert (2022)

## Reflection

No two groups are the same and all have to be tailored to the individuals in the group. For example, group therapy with people who are voice hearers might be textured differently to trauma groups. Some therapists use more experiential work including demonstrations and chair work within the group, more psychodrama and acting out different scenarios, whereas others tend to be more oriented to cognitive processes. Groups might also vary in terms of the culture in which they are embedded. Generally, the more we can embody and enact process the better.

**Acknowledgements** this brief manual is the result of many years of group work. This way of working was first published in 2006 by Gilbert & Procter. Subsequently *many colleagues* have contributed to these ideas, but special thanks go to Drs James Kirby and Nicola Petrocchi who have worked for several years on developing a larger manual with Prof Paul Gilbert OBE that is still under preparation. As noted above, for more insights into group working see Chapters 14-16 of Gilbert, P., & Simos, G. (2022). *Compassion focused therapy: Clinical practice and applications*. Routledge and [www.compassionatemind.co.uk](www.compassionatemind.co.uk)

Please note this guideline is for personal use only and please reference this guideline using Gilbert, et al. (2022). Compassion Focused Group Therapy for People with a Diagnosis of Bipolar Affective Disorder: A Feasibility Study. *Frontiers in Psychology,* 13:841932. doi: 10.3389/fpsyg.2022.841932

© Prof Paul Gilbert (2022)
